# Genome-wide association study identifies novel susceptibility loci for cutaneous squamous cell carcinoma

**DOI:** 10.1038/ncomms12048

**Published:** 2016-07-18

**Authors:** Harvind S. Chahal, Yuan Lin, Katherine J. Ransohoff, David A. Hinds, Wenting Wu, Hong-Ji Dai, Abrar A. Qureshi, Wen-Qing Li, Peter Kraft, Jean Y. Tang, Jiali Han, Kavita Y. Sarin

**Affiliations:** 1 Department of Dermatology, Stanford University School of Medicine, Stanford, California 94305, USA; 2 Department of Epidemiology, Richard M. Fairbanks School of Public Health, Melvin & Bren Simon Cancer Center, Indiana University, Indianapolis, Indiana 46202, USA; 3 23andMe Inc., Mountain View, California 94041, USA; 4 Department of Epidemiology and Biostatistics, Tianjin Medical University Cancer Hospital and Institute, National Clinical Research Center for Cancer, Tianjin & Key Laboratory of Cancer Prevention and Therapy, Tianjin 300060, China; 5 Department of Dermatology, Warren Alpert Medical School, Brown University, Providence, Rhode Island 02903, USA; 6 Department of Epidemiology, School of Public Health, Brown University, Providence, Rhode Island 02903, USA; 7 Channing Division of Network Medicine, Department of Medicine, Brigham and Women's Hospital, Harvard Medical School, Boston, Massachusetts 02115, USA; 8 Department of Epidemiology, Harvard T.H. Chan School of Public Health, Boston, Massachusetts 02115, USA; 9 Department of Biostatistics, Harvard T.H. Chan School of Public Health, Boston, Massachusetts 02115, USA

## Abstract

Cutaneous squamous cell carcinoma represents the second most common cutaneous malignancy, affecting 7–11% of Caucasians in the United States. The genetic determinants of susceptibility to cutaneous squamous cell carcinoma remain largely unknown. Here we report the results of a two-stage genome-wide association study of cutaneous squamous cell carcinoma, totalling 7,404 cases and 292,076 controls. Eleven loci reached genome-wide significance (*P*<5 × 10^−8^) including seven previously confirmed pigmentation-related loci: *MC1R*, *ASIP*, *TYR*, *SLC45A2*, *OCA2*, *IRF4* and *BNC2*. We identify an additional four susceptibility loci: 11q23.3 *CADM1*, a metastasis suppressor gene involved in modifying tumour interaction with cell-mediated immunity; 2p22.3; 7p21.1 *AHR*, the dioxin receptor involved in anti-apoptotic pathways and melanoma progression; and 9q34.3 *SEC16A*, a putative oncogene with roles in secretion and cellular proliferation. These susceptibility loci provide deeper insight into the pathogenesis of squamous cell carcinoma.

## Abstract (web summary)

Cutaneous squamous cell carcinoma is the second most common type of skin cancer. In this genome-wide association study, which includes over 7,000 cases, the authors identify 4 new susceptibility loci for this cancer and also provide independent replication of 9 previously reported susceptibility loci.

## Graphical abstract

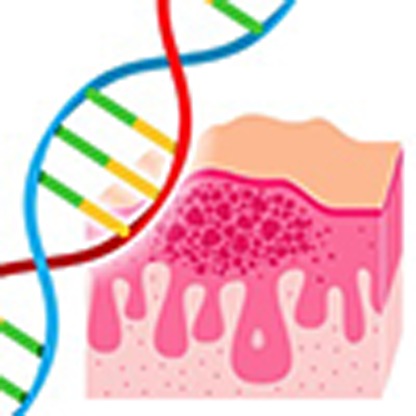

Squamous cell carcinoma (SCC) of the skin represents the second most common cutaneous malignancy, behind only basal cell carcinoma (BCC), with a lifetime incidence of 7–11% in Caucasians in the United States, creating a substantial economic and health burden. In addition, an estimated 6,000 deaths are attributed to SCC annually in the United States^1^. Fair skin, male gender, ultraviolet radiation exposure and prior organ transplant are associated with increased rates of SCC.

In contrast to BCC, few genetic variants have thus far been linked to SCC risk. A recent genome-wide association study (GWAS) from patients within the Kaiser Permanente Healthcare System identified ten SCC susceptibility loci, including six pigmentation loci: *SLC45A2*, *IRF4*, *TYR*, *HERC2*, *DEF8* (16q24, the same locus as *MC1R*) and *RALY*^2^,^3^. Here we report a two-stage genome-wide association meta-analysis for SCC, with a total of 7,404 SCC cases and 292,076 controls from the 23andMe research participant cohort and the Nurses' Health Study/Health Professionals Follow-Up Study (HPFS). Our results provide independent replication for nine of the ten loci from the Kaiser Permanente GWAS and identify four additional novel susceptibility loci for SCC.

## Results

### Genome-wide association study

**Figure 1:**
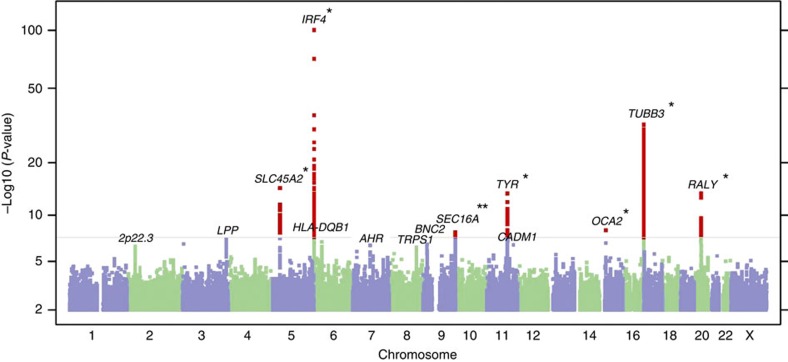
Manhattan plot of stage 1 GWAS analysis of SCC. Total stage 1 GWAS analysis included 6,579 cases and 280,558 controls from the 23andMe cohort. The *y* axis represents log-scaled *P*-values. Loci with smallest *P*<10^−5^ (via logistic regression), of which there are 14 in total, are labelled with the name of the nearest gene. Positions with *P*<5 × 10^−8^ (genome-wide significance) are shown in red. Seven SCC susceptibility loci reached genome-wide significance in stage 1, including six previously reported pigmentation loci at 6p25.3 (*IRF4*), 5p13.2 (*SLC45A2*), 16q24.3 (*MC1R*), 11q14.3 (*TYR*), 20q11.22 (*RALY*-*ASIP*) and 15q13.1 (*OCA2*), which are highlighted by asterisks. The seventh susceptibility locus reaching significance in stage 1, 9q34.3 (*SEC16A*), is novel and not pigmentation related (marked by a double asterisk). Three additional novel susceptibility loci, 2p22.3, 7p21.1 (*AHR*) and 11q23.3 (*CADM1*-*BUD13*), reached genome-wide significance in the overall meta-analysis, as did the previously reported locus 9p22.2 (*BNC2*-*CNTLN*) (Table 2). The remaining three loci in the figure, 3q28 (*LPP*), 6p21.3 (*HLA-DQB1*) and 8q23.3 (*TRPS1*), had *P*<10^−5^ in stage 1 but did not reach genome-wide significance in the meta-analysis.

Stage 1 consisted of a GWAS set encompassing 6,579 self-reported SCC cases and 280,558 controls of European ancestry from the 23andMe research participant cohort (Table 1). A validation study of the 23andMe self-reported data was conducted, which revealed a sensitivity and specificity of 92% and 98%, respectively (Supplementary Table 1). In stage 1, the most significant single-nucleotide polymorphism (SNP) at each locus associated with SCC (*P*<10^−5^) was identified, yielding a list of 14 index SNPs (Fig. 1). Stage 2 consisted of an independent GWAS set of 825 adjudicated SCC cases and 11,518 controls of European ancestry from the Nurses' Health Study and HPFS (Table 1) to confirm the index SNPs identified in stage 1. Subsequently, meta-analysis of stage 1 and stage 2 was performed, encompassing 7,404 SCC cases and 292,076 controls, and identified a total of 11 SNPs reaching genome-wide significance (Table 2, *P*<5 × 10^−8^, logistic regression). Out of the 11 genome-wide significant SNPs, 9 were associated with an increased SCC risk relative to the minor allele, whereas 2 (*SLC45A2* rs35407 and *BNC2* rs10810657) were protective (Table 2). QQ plots, forest plots and misclassification analysis are provided in Supplementary Figs 1–8.

### Confirmation of previously reported loci

Among the 11 genome-wide significant loci identified in this two-stage study (Table 2), 7 were previously reported in the Kaiser GWAS but had not yet been replicated in an external cohort (Table 3, Supplementary Table 2 and Supplementary Fig. 9). Of the seven previously reported loci, six are pigmentation related, whereas the seventh, 9p22.2 *BNC2*, is a putative pigmentation locus^3^. In addition to replicating these seven loci at genome-wide significance, our study replicated two additional loci from the Kaiser GWAS, 3p13 *FOXP1* and 3q28 *TPRG1/TP63* (*P*=4.4 × 10^−3^ and *P*=1.3 × 10^−2^, respectively, by logistic regression) (Table 3 and Supplementary Table 2).

### Genome-wide significant novel susceptibility loci

Our study also identified four novel SCC susceptibility loci (Table 2, Supplementary Table 3 and Supplementary Fig. 10) not previously reported. These four novel susceptibility loci—2p22.3, 7p21.1 (*AHR*), 9q34.3 (*SEC16A*) and 11q23.3 (*CADM1*-*BUD13*)—have not been previously associated with pigmentation phenotypes. Of these four loci, one (*SEC16A*) reached genome-wide significance in stage 1, whereas the other three did so in the combined meta-analysis. Although some loci did not reach statistical significance in stage 2, their 95% confidence intervals (for odds ratios (ORs)) overlapped with corresponding stage 1 confidence intervals. The power to reach *P*<0.05 in stage 2 for all 14 index SNPs is detailed in Supplementary Table 4.

### Heritability of SCC

To measure the proportion of SCC heritability that can be attributed to these SNPs, we calculated the familial relative risk for SCC as outlined by the Cancer Oncological Gene–Environment Study. Overall, 6.3% of familial relative risk for SCC is explained by these 11 loci. Interestingly, although consistent effects for all 7 SNPs were observed across age and gender, larger effect sizes tended to occur in younger cases, perhaps highlighting the increasingly important influence of environmental factors with age (Supplementary Tables 5 and 6, and Supplementary Fig. 11). Further results can be found in Supplementary Tables 7–11 and Supplementary Fig. 12.

## Discussion

### Previously reported loci

The predominance of SCC susceptibility loci associated with pigmentation genes confirms the well-established heritable phenotypic risk of SCC. In our study, these SNPs include the following: rs1805007 (*MC1R* R151C), a red hair allele associated with photosensitivity and increased BCC risk^4^,^5^; rs12203592, which lies within an enhancer of *IRF4* transcription in melanocytes and is associated with increased risk of actinic keratoses (SCC precursors) independent of skin pigmentation^6^,^7^,^8^; rs1126809 (*TYR* R402Q), associated with photosensitivity, tanning and increased risk of BCC and melanoma^9^; rs6059655, intergenic near *RALY*-*ASIP* and associated with facial pigmented spots^10^; and rs35407, in modest linkage disequilibrium with rs16891982 (*SLC45A2* F374L, *r*^2^=0.33, *D*'=1), associated with human pigmentation and melanoma risk^11^,^12^. rs16891982 reached genome-wide significance in stage 1 (*P=*8.6 × 10^−13^, OR=1.65, logistic regression) and in the overall meta-analysis (*P*=1.5 × 10^−11^, OR=1.62, logistic regression) (Supplementary Table 7). We also performed sensitivity analysis on *OCA2* rs1800407 and *IRF4* rs12203592 using high imputation quality subsets and directly genotyped data sets (Supplementary Table 8)^13^.

In addition to these confirmed pigmentation loci, rs10810657 at 9p22.2 reached genome-wide significance in the overall meta-analysis (*P*=1.4 × 10^−8^, OR=0.90, logistic regression). rs10810657 lies ∼13 kb upstream of the basonuclin 2 (*BNC2*) transcription start site in a potential enhancer in foreskin melanocytes and other cell types^8^. This SNP is in linkage disequilibrium with rs12350739 (*r*^2^=0.90, *D*′=1.00), which resides in an enhancer element regulating *BNC2* transcription in human melanocytes^14^. BNC2 is a DNA-binding zinc-finger protein thought to act as both a messenger RNA-processing enzyme and a transcription factor^14^. *BNC2* is expressed in melanocytes and, to a lesser extent, keratinocytes, with higher expression levels corresponding to darker skin pigmentation in human skin tissue analysis. Variants in the *BNC2* locus have recently been associated with skin colour, freckling and age-related pigmentation spots in Europeans, in addition to SCC^3^,^10^,^15^. The association and linkage disequilibrium results for these SNPs are listed in Supplementary Tables 9 and 10.

### Novel susceptibility loci

We also identified four novel SNPs associated with SCC. rs57994353 at 9q34.3 (*P*=7.5 × 10^−9^, OR=1.12, logistic regression) resides within an intron of *SEC16A* in tight linkage disequilibrium with rs3812594 *SEC16A* R1039C (*r*^2^=0.90, *D*'=1)^8^. SEC16A is a cytosolic scaffold protein that acts at endoplasmic reticulum exit sites to facilitate vesicle formation and export^16^. Production of this protein is regulated by various growth factors, with higher levels of SEC16A corresponding to increased secretion and cellular proliferation^17^. Overexpression of SEC16A has been observed in colonic adenocarcinoma samples^17^.

Another novel SCC risk variant, rs74899442 at 11q23.3, reached genome-wide significance in the overall meta-analysis (*P*=8.7 × 10^−9^, OR=2.13, logistic regression). This SNP lies in an intergenic region 515 kb upstream of *CADM1*, which encodes a single-pass transmembrane protein involved in cell–cell adhesion and signal transduction^18^,^19^,^20^. *CADM1* is also a putative tumour suppressor in many human carcinomas, including SCC, as it is frequently downregulated in these tissues via promoter methylation^21^,^22^,^23^. Notably, *CADM1* expression levels are associated with survival in SCC patients. In a study of 87 patients with SCC, those with decreased CADM1 expression had significantly shorter median survival (36 versus 54 months)^23^. Conversely, overexpression of CADM1 in SCC cells suppresses cell proliferation and promotes apoptosis^23^. In light of these recent findings from various studies, our results provide further evidence that *CADM1* may play a role in SCC development.

The third and fourth novel susceptibility variants rs192481803 and rs117132860 reached genome-wide significance in the overall meta-analysis. rs192481803 at 2p22.23 (*P*=4.5 × 10^−8^, OR=1.90, logistic regression) lies within a long non-coding RNA AC012593.1 of unknown function^8^. rs117132860 (*P=*3.6 × 10^−8^, OR=1.48, logistic regression), located at 7p21.1, is situated within a DNAse hypersensitivity site 203 kb upstream of *AHR*, the aryl hydrocarbon receptor, and has predicted enhancer activity in multiple tissues including keratinocytes. AHR is a widely expressed transcription factor that is involved in drug metabolism and cellular proliferation, although its exact role is tissue dependent and oftentimes paradoxical^24^,^25^,^26^. Interestingly, in the context of keratinocytes, ultraviolet radiation activates AHR and triggers a molecular cascade (known as the AHR-E2F1-CHK1 axis) that ultimately inhibits apoptosis^24^. This inhibition allows ultraviolet-induced DNA damage and reactive oxygen species to accumulate within keratinocytes; hence, overexpression of *AHR* may contribute to the development of non-melanoma skin cancers.

### Suggestive novel susceptibility loci

We also provide evidence of additional SCC susceptibility loci. Three loci, 6p21.32 (rs28993540), 3q28 (rs11715549) and 8q23.3 (rs199816436), with high imputation quality (*R*^2^>0.9) were associated with SCC risk in stage 1 (*P*<10^−5^) but did not reach *P*<0.05 in stage 2, or *P*<5 × 10^−8^ in the overall meta-analysis (Supplementary Table 11 and Supplementary Fig. 12). rs28993540 lies 35 kb upstream of *HLA-DQB1*. Transplant and other immunosuppressed patients are at significantly increased risk of SCCs suggesting a role for T-cell-mediated protection against SCC^27^. *HLA-DQB1* alleles have been associated with risk of squamous cell cervical cancer and may function by altering the efficiency of the T-cell-mediated immune response to HPV antigens^28^. rs11715549 resides in a potential enhancer in keratinocytes within *LPP*, a gene involved in epithelial cell motility and cell–cell adhesion. rs11715549 is in tight linkage disequilibrium with SNPs associated with vitiligo (rs9851967, *r*^2^=0.93), celiac disease (rs1464510, *r*^2^=0.74) and allergy (rs9860547, *r*^2^=0.87)^8^,^29^. rs199816436 lies inside an intron in *TRPS1*, a transcription factor that represses *GATA*-regulated genes and binds to a dynein light chain protein. rs199816436 is tightly correlated with rs10808475 (*r*^2^=0.85) and is a *TRPS1* expression quantitative trait locus (eQTL) in liver^30^. Defects in *TRPS1* lead to trichorhinophalangeal syndrome, a genetic syndrome characterized by coarse facies, brittle hair and skeletal defects. Although falling short of genome-wide significance, this evidence is nonetheless suggestive of an association between these loci and SCC that merits further investigation.

This two-stage meta-analysis provides the first independent replication of nine of ten previously reported SCC susceptibility loci and identifies four novel susceptibility loci. In addition, this large-scale GWAS demonstrates the power of consumer self-reported data from internet platforms as a resource for discovering cancer susceptibility loci, with results consistent with studies using adjudicated cancer data.

## Methods

### Stage 1 study design and population

23andMe (Mountain View, CA), a genetics company, provided free access to anonymized genetic and phenotypic information for stage 1 of this GWAS. All information came from 23andMe research participants who provided informed consent to take part in this research, in accord with 23andMe's human subjects protocol (reviewed and approved by Ethical and Independent Review Services, an Association for the Accreditation of Human Research Protection Program (AAHRPP)-accredited Institutional Review Board (IRB)). 23andMe gathers genetic information by genotyping sample material provided by its customers; phenotypic information is collected via customer responses to online surveys. Inclusion and exclusion criteria are discussed below.

### Stage 1 genome-wide association analysis

Association analysis for stage 1 was performed using logistic regression, assuming an additive model for allelic effects. The analysis was adjusted for age, sex and population stratification (using the first five principal components), generating the following model:


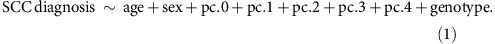


The association test *P*-value was computed using a likelihood ratio test. Results for the X chromosome were computed similarly, with male genotypes coded as if they were homozygous diploid for the observed allele. In addition, test statistics were adjusted for genomic control, to correct for residual population stratification persisting after principal component analysis; the genomic control inflation factor was 1.085 (computed from the median *P*-value for results that passed quality control). Regions of interest were defined by identifying SNPs with *P*<10^−5^, then grouping these into intervals separated by gaps of at least 250 kb and choosing the SNP with smallest *P* within each interval.

### Stage 1 genotyping and quality control

Samples were genotyped on one of four genotyping platforms. The V1 and V2 platforms were variants of the Illumina HumanHap550+ BeadChip, including about 25,000 custom SNPs selected by 23andMe, with a total of about 560,000 SNPs. The V3 platform was based on the Illumina OmniExpress+ BeadChip, with custom content to improve the overlap with our V2 array, with a total of ∼950,000 SNPs. The V4 platform in current use is a fully custom array, including a lower redundancy subset of V2 and V3 SNPs with additional coverage of lower-frequency coding variation, and ∼570,000 SNPs. Samples that failed to reach 98.5% call rate were re-analysed. Individuals whose analyses failed repeatedly were re-contacted by 23andMe customer service to provide additional samples, as is done for all 23andMe customers.

Individuals were only included if they had >97% European ancestry, as determined through an analysis of local ancestry^31^. Briefly, this analysis first partitions phased genomic data into short windows of ∼100 SNPs. Within each window, a support vector machine is used to classify individual haplotypes into one of 31 reference populations. The support vector machine classifications are then fed into a hidden Markov model (HMM) that accounts for switch errors and incorrect assignments, and gives probabilities for each reference population in each window. Finally, simulated admixed individuals are used to recalibrate the HMM probabilities so that the reported assignments are consistent with the simulated admixture proportions. The reference population data are derived from public data sets (the Human Genome Diversity Project, HapMap and 1000 Genomes) and from 23andMe research participants who have reported having four grandparents from the same country.

A maximal set of unrelated individuals was chosen for each analysis using a segmental identity-by-descent (IBD) estimation algorithm^32^. Individuals were defined as related if they shared more than 700 cM IBD, including regions where the two individuals share either one or both genomic segments identical-by-descent. This level of relatedness (roughly 20% of the genome) corresponds approximately to the minimal expected sharing between first cousins in an outbred population.

Participant genotype data were imputed against the March 2012 ‘v3' release of 1000 Genomes reference haplotypes^33^. Data for each genotyping platform were phased and imputed separately. First, Beagle^34^ (version 3.3.1) was used to phase batches of 8,000–9,000 individuals across chromosomal segments of no more than 10,000 genotyped SNPs, with overlaps of 200 SNPs. SNPs with Hardy–Weinberg equilibrium *P*<10^−20^, call rate <95%, or with large allele frequency discrepancies compared with European 1000 Genomes reference data were excluded. Frequency discrepancies were identified by computing a 2 × 2 table of allele counts for European 1000 Genomes samples and 2,000 randomly sampled 23andMe research participants with European ancestry, and identifying SNPs with a *χ*^2^-*P*<10^−15^. Each phased segment was imputed against all-ethnicity 1000 Genomes haplotypes (excluding monomorphic and singleton sites) using Minimac2 (ref. 35), using 5 rounds and 200 states for parameter estimation.

For the non-pseudoautosomal region of the X chromosome, males and females were phased together in segments, treating the males as already phased; the pseudoautosomal regions were phased separately. Males and females were then imputed together using minimac, as with the autosomes, treating males as homozygous pseudo-diploids for the non-pseudoautosomal region.

For quality control of genotyped GWAS results, SNPs that were only genotyped on the ‘V1' platform were flagged due to small sample size, and SNPs on chrM or chrY, because many of these are not currently called reliably. Using trio data, SNPs that failed a test for parent–offspring transmission were also flagged; specifically, the child's allele count was regressed against the mean parental allele count and SNPs with fitted *β*<0.6 and *P*<10^−20^ for a test of *β*<1 were flagged. SNPs with a Hardy–Weinberg *P*<10^−20^ in Europeans, or a call rate of <90%, were also flagged. Genotyped SNPs were also tested for genotype date effects and SNPs with *P*<10^−50^ by analysis of variance of SNP genotypes against a factor dividing genotyping date into 20 roughly equal-sized buckets were flagged.

For imputed GWAS results, SNPs with avg.rsq<0.5 or min.rsq<0.3 in any imputation batch were flagged, as well as SNPs that had strong evidence of an imputation batch effect. The batch effect test was an F-test from an analysis of variance of the SNP dosages against a factor representing imputation batch; results with *P*<10^−50^ were flagged. Before GWAS, the largest subset of the data passing these criteria was identified for each SNP, based on their original genotyping platform—either v2+v3+v4, v3+v4, v3 or v4 only—and association test results were computed for whatever was the largest passing set. As a result, there were no imputed results for SNPs that failed these filters.

When choosing between imputed and genotyped GWAS results, if either the imputed test passed quality control, or a genotyped test was unavailable, the imputed result was reported; otherwise, the genotyped result was reported. For tests using imputed data, imputed dosages were used rather than best-guess genotypes.

Across all results, logistic regression results that did not converge due to complete separation, identified by abs(effect)>10 or stderr>10 on the log odds scale, were flagged. Linear regression results for SNPs with minor allele frequency <0.1% were also flagged, as tests of low-frequency variants can be sensitive to violations of the regression assumption of normally distributed residuals. This methodology has been applied in prior GWAS studies^36^,^37^,^38^.

### Stage 1 phenotype categorization

23andMe identified SCC cases by using research participants' self-reported answers to online questionnaires. Subjects who answered ‘Yes' and/or selected SCC from a dropdown menu in response to at least one of the following questions were defined as cases: ‘Have you ever been diagnosed by a doctor with squamous cell carcinoma?' ‘What type of skin cancer did you have? Please check all that apply.' ‘What type of skin cancer or cancers have you been diagnosed with? Please check all that apply.' ‘Have you ever been diagnosed with squamous cell carcinoma?' ‘Have you ever been diagnosed or treated for any of the following conditions?' Controls were defined as subjects who answered ‘No' and did not select SCC from any relevant dropdown menus. In addition, subjects who answered ‘No' to at least one of the following questions (and ‘Yes' to none) were defined as controls: ‘Have you ever been diagnosed with cancer, including skin cancer or cancerous moles?' ‘Has a doctor ever told you that you have a type of cancer?' ‘Have you ever been diagnosed or treated with any of the following conditions?' Among the samples with imputed genotypes, 23andMe has 6,579 SCC cases and 280,558 controls.

### Sensitivity and specificity of stage 1 self-reported data

To assess the validity of self-reported phenotypic data in stage 1, 23andMe surveys (pertaining to skin cancer history and pigmentation) were randomly administered to 188 patients seen in Stanford outpatient clinics. The survey answers were then compared with medical records to assess for accuracy with respect to SCC diagnosis, to determine the sensitivity and specificity of the survey responses. *P*-values were determined using *χ*^2^-analysis. This sub-study was approved by the Stanford University Institutional Review Board with a waiver of documentation of consent.

### Stage 2 study design and population

The Nurses' Health Study was established in 1976, when 121,700 female registered nurses between the ages of 30 and 55 years residing in 11 larger US states completed and returned an initial self-administered questionnaire on their medical histories and baseline health-related exposures. Biennial questionnaires with collection of exposure information on risk factors have been collected prospectively. Every 2 years, along with exposures, outcome data with appropriate follow-up of reported disease events are collected. Overall, follow-up has been high; after more than 20 years, ∼90% of participants continue to complete questionnaires. From May 1989 through September 1990, we collected blood samples from 32,826 participants in the NHS. Information on SCC development was first collected in the 1984 questionnaire.

The HPFS was established in 1986 when 51,529 men from all 50 US states in health professions (dentists, pharmacists, optometrists, osteopath physicians, podiatrists and veterinarians) aged 40–75 years answered a detailed mailed questionnaire. The average follow-up rate for this cohort over 10 years is >90%. On each biennial questionnaire, we obtained disease- and health-related information. Between 1993 and 1994, 18,159 study participants provided blood samples by overnight courier. Information on SCC development was first collected in the 1986 questionnaire.

The protocol for this study was approved by the Institutional Review Board at Brigham and Women's Hospital and the Harvard School of Public Health. All of the participants provided informed consent.

### Stage 2 genotyping and quality control

There were 18 GWAS data sets from the NHS and HPFS as nested case–control studies with cleaned genotype data available. We combined these data sets into three complied data sets based on their genotype platform type: Affymetrix, Illumina HumanHap series or Illumina Omni Express. The Affymetrix data set comprises data on the Affy 6.0 platform (NHS-type 2 diabetes, NHS-coronary heart disease, HPFS-type 2 diabetes and HPFS-coronary heart disease). The Illumia HumanHap data set comprises several platforms: Illumina 550 K (NHS-breast cancer, NHS-Pancreas cancer and HPFS-pancreas cancer), Illumina 610Q (NHS-kidney stone, HPFS-kidney stone and HPFS-prostate cancer) and Illumina 660 (NHS-glaucoma and HPFS-glaucoma). The Illumina Omni Express data set contained only studies genotyped on the Omni Express platform (NHS-endometrial cancer, NHS-colon cancer, NHS-mammographic density, NHS-gout, HPFS-colon and HPFS-gout).

We combined the individual data sets that were genotyped on the same platform, removing any SNPs that were not in all studies and with a missing call rate >5%, and flipping strands, where appropriate, to create a final compiled data set. This resulted in 668,283 SNPs in the Affymetrix data set, 459,999 SNPs in the Illumina HumanHap data set and 565,810 SNPs in the Illumina Omni Express data set. Analyses were restricted to subjects with self-reported European ancestry. Genetic principal components were calculated using sets of independent SNPs (12,000–33,000 SNPs depending on platform). Subjects who did not cluster with other self-identified Europeans based on the top five principal components were also excluded.

We then ran a pairwise IBD analysis for each combined data set to detect duplicate and related individuals based on resulting *Z*-scores. If 0≤Z0≤0.1, 0≤Z1≤0.1 and 0.9≤2≤1.1, then a pair was flagged as being identical twins or duplicates. Pairs were considered full siblings if 0.17≤Z0≤0.33, 0.4≤Z1≤0.6 and 0.17≤Z2≤0.33. Half siblings or avunculars were defined as having 0.4≤Z1≤0.6 and 0≤Z2≤0.1. Some of the duplicates flagged in this step were expected, having been genotyped in multiple data sets and hence having the same cohort identifications (IDs). In this case, one of each pair was randomly chosen for removal from the data set. Instances where pairs were flagged as unexpected duplicates with the different cohort IDs, but pairwise genotype concordance rate>0.999, resulted in removal of both individuals from the pair. Related individuals (full sibs and half sibs/avunculars) were not removed from the final data sets. In the Affymetrix data set 167 individuals were removed, because they were duplicates or were flagged for removal from secondary genotype data cleaning, leaving a total of 8,065 individuals. Of the 6,894 individuals originally in the Illumina data set 107 were removed, because they were duplicates or flagged for removal in the genotyping step, leaving 6,787 IDs. In addition, eight pairs of individuals were flagged as related. In the Omni express data set, there were 5,956 individuals at the start with 39 IDs to remove, leaving 5,917 IDs and 5 pairs of related IDs.

After removing duplicate IDs and flagging related pairs of IDs, we used eigenstrat to run principal component analysis on each compiled data set, removing one member from each flagged pair of related individuals. For Affymetrix and Illumina HumanHap, we used ∼12,000 SNPs that were filtered, to ensure low pairwise linkage disequilibrium (LD)^39^. For the OmniExpress data set we used ∼33,000 SNPs that were similarly filtered. We plotted the top eigenvectors using R and examined the plots for outliers.

Finally, as a quality-control check, we ran logistic regression analyses using each individual study's controls as ‘cases' and the rest of the studies controls as ‘controls.' For example, in the Illumina Omni Express data set, we ran regressions of NHS-gout controls considered as ‘cases' versus the HPFS-gout, NHS-endometrial cancer, NHS-colon cancer, NHS-mammographic density and HPFS-colon cancer. We then ran regressions with each of the other study controls as ‘cases' versus all of the rest of the controls. We looked for *P*-values of genome-wide significance (*P*<10^−8^) and examined QQ plots to determine whether any SNPs were flagged as significant where no SNPs should have been significant. In the Affymetrix data set, 100 SNPs were flagged and removed. In the Illumina HumanHap data set, eight SNPs had *P*<10^−8^ in any of the quality control (QC) regressions and were removed. No SNPs in the Illumina Omni Express data set had *P*-values<10^−8^; hence, no additional SNPs needed to be removed. After the data sets were combined and appropriate SNP and ID filters applied, the complied data sets were imputed.

Using combined GWAS genotypes on each genotyping platform and the 1000 Genomes Project ALL Phase I Integrated Release Version 3 Haplotypes excluding monomorphic and singleton sites (2010–11 data freeze, 2012-03-14 haplotypes) as reference panel, we imputed the genotypes of markers in the 1000 Genomes Project for 8,065 samples in Affymetrix data set, 6,787 samples in Illumina HumanHap data set and 5,917 samples in Illumina Omni Express data set.

SNP genotypes were imputed in three steps. First, genotypes on each chromosome were split into chunks, to facilitate windowed imputation in parallel using ChunkChromosome (v.2011-08-05) (http://genome.sph.umich.edu/wiki/ChunkChromosome). Next, each chunk of chromosome was phased using MACH (v.1.0.18.c) (http://www.sph.umich.edu/csg/abecasis/MaCH/index.html). In the final step, Minimac (v.2012-08-15) (http://genome.sph.umich.edu/wiki/Minimac) was used to impute the phased genotypes to ∼31 million markers in the 1000 Genomes Project^35^.

### Stage 2 phenotype categorization

Participants in both NHS and HPFS cohorts reported new SCC diagnosis biennially. With their permission, medical records were obtained and reviewed to confirm their self-reported diagnosis. Eligible cases in the NHS and HPFS consisted of participants with pathologically confirmed invasive SCC, diagnosed any time after baseline up to the 2012 follow-up cycle for both cohorts. Samples free of diagnosis of SCC were controls in this study. In the three compiled data sets, samples without information on SCC diagnosis were excluded. Among the samples with imputed genotypes, we have 367 SCC cases and 5,453 controls in Affymetrix data set, 220 SCC cases and 2,901 controls in Illumina HumanHap data set and 238 SCC cases and 3,164 controls in Illumina Omni Express data set— 825 SCC cases and 11,518 controls in total.

### Stage 2 genome-wide association analysis

We used ProbABEL software to test the GWAS association between minor allele counts and SCC risk using imputed dosage data. We performed logistic regression analysis under an additive model with adjustment for age, sex, basal cell carcinoma (BCC) history and the first five principal components, generating the following model:


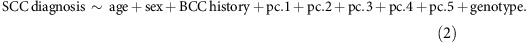


These principal components were calculated for all individuals on the basis of ∼10,000 unlinked markers using the EIGENSTRAT software^40^. Associations in each component GWAS set (Affymetrix, Illumina HumanHap series and Illumina Omni Express) were combined in an inverse-variance-weighted meta-analysis using the METAL software.

### Meta-analysis

For all 14 index SNPs, the same meta-analysis was conducted to combine stage 1 and stage 2. Heterogeneity of per-SNP effect sizes in studies contributing to the stage 1, stage 2 and overall meta-analyses was assessed and fixed-effects meta-analysis was conducted. All *R*^2^ and *D*' values between individual SNPs were calculated based on the 1000 Genomes Pilot 1 data set, CEU Population (http://www.broadinstitute.org/mpg/snap/ldsearchpw.php)^41^.

### Proportion of familial relative risk

We have used the formula for calculating the proportion of familial relative risk (FRR) as outlined by the Cancer Oncological Gene-environment Study (http://www.nature.com/icogs/primer/common-variation-and-heritability-estimates-for-breast-ovarian-and-prostate-cancers/#70) as described previously^42^. The ORs derived from our meta-analysis of stage 1 and stage 2 are assumed to be relative risks. We estimated the proportion of the FRR explained by each SNP (FRR_snp_) as:


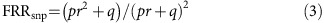


Here, the risk allele and alternative allele frequencies are *p* and *q*, respectively, and *r* is the OR for the risk allele. Allele frequencies were derived from the 1000 Genomes Project European population data. Assuming that the loci combine multiplicatively and are not in linkage disequilibrium, the combined effect of all loci is given by:


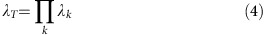


Here, the product is across all loci. The proportion of the familial relative risk attributable to the SNPs, on a log scale, is then given by:


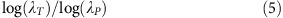


In this equation, *λ*_*P*_ is the familial relative risk observed in epidemiological studies. *λ*_*P*_ is fourfold for SCC^43^.

### Regulatory function of novel variants

For each novel SCC susceptibility variant, we searched for evidence of regulatory function using HaploReg version 4 (refs 8, 44) (http://www.broadinstitute.org/mammals/haploreg/haploreg.php)^45^. We queried each rsID and extracted data from ENCODE Project Consortium 2011–2012 on closest annotated gene, chromatin immunoprecipitation-sequencing transcription factor binding, DNaseI hypersensitivity sites, and enhancer and promoter chromatin segmentation states^46^,^47^,^48^. Data were also extracted from Roadmap Epigenomics Consortium 2015 on enhancer and promoter chromatin segmentation states, specifically using the following states: 15-state HMM, 25-state HMM, H3K4me1, H3K4me3, H3K27ac and H3K9ac^49^. We particularly focused on the enhancer and promoter annotations that referenced normal human epidermal keratinocytes and primary foreskin keratinocytes. Finally, we used HaploReg v4 to extract eQTL data for each variant, as version 4 is updated with *cis* eQTL data from the GTEx pilot analysis and many other studies^50^. We made special note of variants that were eQTLs in skin tissue.

### Power calculations

Power was computed according to Freidlin *et al*.^51^. To account for misclassification, expected genotype frequencies in study cases were replaced with a mixture of genotype frequencies in true cases and in true controls. Power was plotted as a function of OR for detecting a variant with minor allele frequency 0.1, based on the GWAS sample size and with hypothetical misclassification rates of 0, 10 and 20% (where the specified fraction of study cases are misclassified controls).

### Data availability

GWAS data from the 23andMe research cohort and Nurses' Health Study have not been deposited in public repositories, as consent for this was not obtained in the study protocols. The precomputed rankings and *P*-values for SNPs included in the stage 1 GWAS are available upon request by contacting D.A.H. at dhinds@23andMe.com. Precomputed rankings and *P*-values for the top 10,000 SNPs included in the stage 2 GWAS are freely available by contacting www.channing.harvard.edu/nhs. Any additional data (beyond those included in the main text and Supplementary Information) that support the findings of this study are available from the corresponding author upon request.

## Additional information

**How to cite this article**: Chahal, H. *et al*. Genome-wide association study identifies novel susceptibility loci for cutaneous squamous cell carcinoma. *Nat. Commun.* 7:12048 doi: 10.1038/ncomms12048 (2016).

## Supplementary Material

Supplementary Information Supplementary Figures 1-12, Supplementary Tables 1-11 and Supplementary References.

## Figures and Tables

**Table 1:** Gender and age of cases and controls from each stage of GWAS.

**Status**	***n*** **(%)**	**Male (%)**	**Age<31 years**	**Age 30–45 years**	**Age 46–60 years**	**Age>60 years**
23andMe (Stage 1, *n*=287,137)						
Cases	6,579 (2.3)	3,510 (53)	12 (0.18)	199 (3)	1,263 (19)	5,105 (78)
Controls	280,558 (97.7)	151,588 (54)	39,838 (14)	83,780 (30)	76,833 (27)	80,107 (28)
						
Harvard (Stage 2, *n*=12,343)						
Affy						
Cases	367 (6.3)	183 (49.9)	0 (0.0)	91 (24.8)	213 (58.0)	63 (17.2)
Controls	5,453 (93.7)	2,412 (44.2)	0 (0.0)	1,952 (36.8)	2,729 (50.0)	772 (14.2)
Illumina						
Cases	220 (7.0)	91 (41.4)	0 (0.0)	61 (27.7)	125 (56.8)	34 (15.5)
Controls	2,901 (93.0)	232 (8.0)	0 (0.0)	1,366 (47.1)	1,434 (49.4)	101 (3.5)
Omni						
Cases	238 (7.0)	102 (42.9)	0 (0.0)	72 (30.2)	137 (57.6)	29 (12.2)
Controls	3,164 (93.0)	803 (25.4)	0 (0.0)	1,401 (44.3)	1,488 (47.0)	275 (8.7)
All, Stage 2						
Cases	825 (6.7)	376 (45.6)	0 (0.0)	224 (27.1)	475 (57.6)	126 (15.3)
Controls	11518 (93.3)	3447 (29.9)	0 (0.0)	4719 (41.0)	5651 (49.1)	1148 (9.9)
						
Combined meta-analysis (*n*=29,9480)						
Cases	7,404 (2.5)	3,886 (52)	12 (0.16)	423 (5.7)	1,738 (23)	5,231 (71)
Controls	29,2076 (97.5)	155,035 (53)	39,838 (14)	88,499 (30)	82,484 (28)	81,255 (28)

GWAS, genome-wide association study.

Counts and percentages for cases and controls (*n* (%)) are listed above, stratified by stage of GWAS. We also report number and percentage of male subjects, subjects with age <31 years, subjects with age 31–45 years, subjects with age 46–60 years and subjects with age >60 years. Stage 2 cases and controls are further subdivided based on platform used for genotyping.

**Table 2:** Eleven loci reaching genome-wide significance in two-stage GWAS of SCC.

					**Stage 1**		**Stage 2**		**Meta-analysis**	
**SNP**	**Region**	**Gene**	**Major/minor**	**MAF (avg imp** ***r***^**2**^)	**OR**	***P*****-values**	**OR**	***P*****-values**	**OR**	***P*****-values**
rs12203592	6p25.3	*IRF4*	C/T	0.17 (0.99)	1.62	3.0 × 10^-101^	1.60	3.1 × 10^-6^	1.62	2.9 × 10^-111^
rs1805007	16q24.3	*MC1R*	C/T	0.07 (1.0)	1.45	5.8 × 10^-33^	1.64	4.9 × 10^-5^	1.46	8.5 × 10^-39^
rs35407	5p13.2	*SLC45A2*	G/A	0.04 (0.98)	0.59	3.6 × 10^-15^	0.62	5.5 × 10^-2^	0.59	1.3 × 10^-13^
rs1126809	11q14.3	*TYR*	G/A	0.28 (0.99)	1.17	3.5 × 10^-14^	1.08	3.3 × 10^-1^	1.16	3.0 × 10^-14^
rs6059655	20q11.22	*RALY-ASIP*	G/A	0.07 (0.99)	1.28	3.6 × 10^-14^	1.08	5.4 × 10^-1^	1.27	2.5 × 10^-14^
rs1800407	15q13.1	*OCA2*	C/T	0.07 (1.0)	1.21	8.0 × 10^-9^	0.97	8.3 × 10^-1^	1.20	8.9 × 10^-9^
rs57994353*	9q34.3	*SEC16A*	T/C	0.30 (0.99)	1.12	1.4 × 10^-8^	1.05	4.7 × 10^-1^	1.12	7.5 × 10^-9^
rs10810657	9p22.2	*BNC2, CNTLN*	A/T	0.41 (0.98)	0.91	2.4 × 10^-7^	0.83	1.2 × 10^-2^	0.90	1.4 × 10^-8^
rs74899442*	11q23.3	*CADM1, BUD13*	T/C	0.01 (0.62)	2.12	3.0 × 10^-7^	2.42	1.8 × 10^-1^	2.13	8.7 × 10^-9^
rs192481803*	2p22.3	*unknown*	C/T	0.01 (0.58)	1.92	4.5 × 10^-7^	1.51	4.3 × 10^-1^	1.90	4.5 × 10^-8^
rs117132860*	7p21.1	*AHR*	G/A	0.02 (0.52)	1.46	7.2 × 10^-7^	1.80	4.0 × 10^-2^	1.48	3.6 × 10^-8^

Avg imp, average imputation; GWAS, genome-wide association study; MAF, minor allele frequency; OR, odds ratio; SCC, squamous cell carcinoma; SNP, single-nucleotide polymorphism.

SNPs that met genome-wide significance (*P*<5 × 10^−8^) via logistic regression in stage 1 and/or overall meta-analysis are listed. In addition, we report genetic locus, nearest genes, major allele, minor allele, MAF as calculated from stage 1 data, avg imp *r*^2^ (a measure of imputation quality) for stage 1 and OR with *P*-value for each stage, calculated with respect to the minor allele. In stage 1, we analysed 6,579 SCC cases and 280,558 controls of European ancestry in the United States. In stage 2, we analysed 825 SCC cases and 11,518 controls of European ancestry in the United States. We then combined the data from these 2 stages (which resulted in 7,404 SCC cases and 292,076 controls) and performed fixed-effect meta-analysis. Statistics for effect heterogeneity (*P*_*het*_ and *I*^2^) are included in Supplementary Tables 2 and 3. Asterisks highlight novel loci.

**Table 3:** Replication of ten previously reported SCC-associated loci.

					**Stage 1**		**Stage 2**		**Meta-analysis**		**Prior GWAS**	
**SNP**	**Region**	**Gene**	**Major/minor**	**MAF (avg imp** ***r***^**2**^)	***P*****-values**	**OR**	***P*****-values**	**OR**	***P*****-values**	**OR**	***P*****-values**	**OR**
rs12203592	6p25.3	*IRF4*	C/T	0.17 (0.99)	3.0 × 10^-101^	1.62	2.1 × 10^-5^	1.56	1.9 × 10^-110^	1.62	8.3 × 10^-97^	1.56
rs1805007	16q24.3	*MC1R*	C/T	0.07 (1.0)	5.8 × 10^-33^	1.45	9.5 × 10^-5^	1.64	1.5 × 10^-38^	1.46	1.8 × 10^-44^	1.33
rs6059655	20q11.22	*RALY-ASIP*	G/A	0.07 (0.99)	3.6 × 10^-14^	1.28	8.2 × 10^-1^	1.03	3.2 × 10^-14^	1.26	9.0 × 10^-21^	1.32
rs35407	5p13.2	*SLC45A2*	G/A	0.04 (0.98)	3.6 × 10^-15^	0.59	3.6 × 10^-2^	0.58	4.2 × 10^-14^	0.59	2.8 × 10^-28^	0.52
rs1126809	11q14.3	*TYR*	G/A	0.28 (0.99)	3.5 × 10^-14^	1.17	8.1 × 10^-1^	1.02	8.3 × 10^-14^	1.16	2.2 × 10^-20^	1.19
rs1800407	15q13.1	*OCA2*	C/T	0.07 (1.0)	8.0 × 10^-9^	1.21	8.9 × 10^-1^	0.98	7.7 × 10^-9^	1.20	3.3 × 10^-9^	0.88
rs10810657	9p22.2	*BNC2, CNTLN*	A/T	0.41 (0.98)	2.4 × 10^-7^	0.91	7.9 × 10^-3^	0.82	1.2 × 10^-8^	0.90	8.2 × 10^-9^	0.9
rs62246017	3p13*	*FOXP1*	G/A	0.33 (0.85)	3.2 × 10^-3^	1.06	9.6 × 10^-1^	1.00	4.4 × 10^-3^	1.06	1.2 × 10^-8^	1.11
rs6791479	3q28*	*TPRG1/TP63*	A/T	0.43 (0.99)	7.0 × 10^-2^	1.03	3.5 × 10^-3^	1.23	1.3 × 10^-2^	1.05	1.5 × 10^-11^	1.13
rs4455710	6p21*	*HLA-DQA1*	C/T	—	—	—	—	—	—	—	1.9 × 10^-18^	1.17

Avg imp, average imputation; GWAS, genome-wide association study; MAF, minor allele frequency; OR, odds ratio; SCC, squamous cell carcinoma; SNP, single-nucleotide polymorphism.

Ten loci previously reported as associated with SCC via prior GWAS (*P*<5 × 10^−8^) are listed, the first seven of which also reached genome-wide significance in this study. Of the remaining three loci (asterisks), two (3p13 and 3q28) reached nominal significance (*P*<0.05) in this study. In addition, we report genetic locus, nearest genes, major allele, minor allele, MAF as calculated from stage 1 data, avg imp *r*^2^ (a measure of imputation quality) for stage 1 and OR with *P*-value for each stage, calculated with respect to the minor allele. The right-most two columns list *P*-value and OR from prior GWAS^3^ for each locus, relative to the minor allele.
