# Supplementary material for: Genome-wide association study identifies novel susceptibility loci for cutaneous squamous cell carcinoma
Source: Nat Commun. 2016 Jul 18;7:12048. doi: 10.1038/ncomms12048 (PMC4960294; doi:10.1038/ncomms12048)
Supplement: Supplementary Information — Supplementary Figures 1-12, Supplementary Tables 1-11 and Supplementary References. [file ncomms12048-s1.pdf]

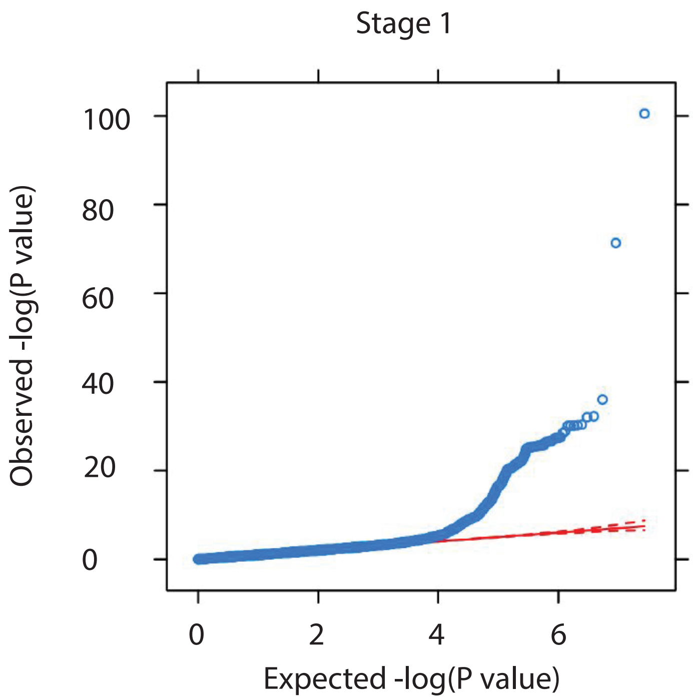

Supplementary Figure 1. QQ plot of observed versus expected quantiles for the SCC GWAS stage 1 P values (generated via logistic regression), plotted on a log scale. The null hypothesis states that the expected distribution of P values is uniform. Here, the observed P values follow the null distribution for large P values (P>0.01) but then diverge for small P values. The solid red line has a slope 1 and the dashed red lines represent a 95% confidence interval, assuming the test results are independent. The test statistics in the plot have already been adjusted for genomic control.

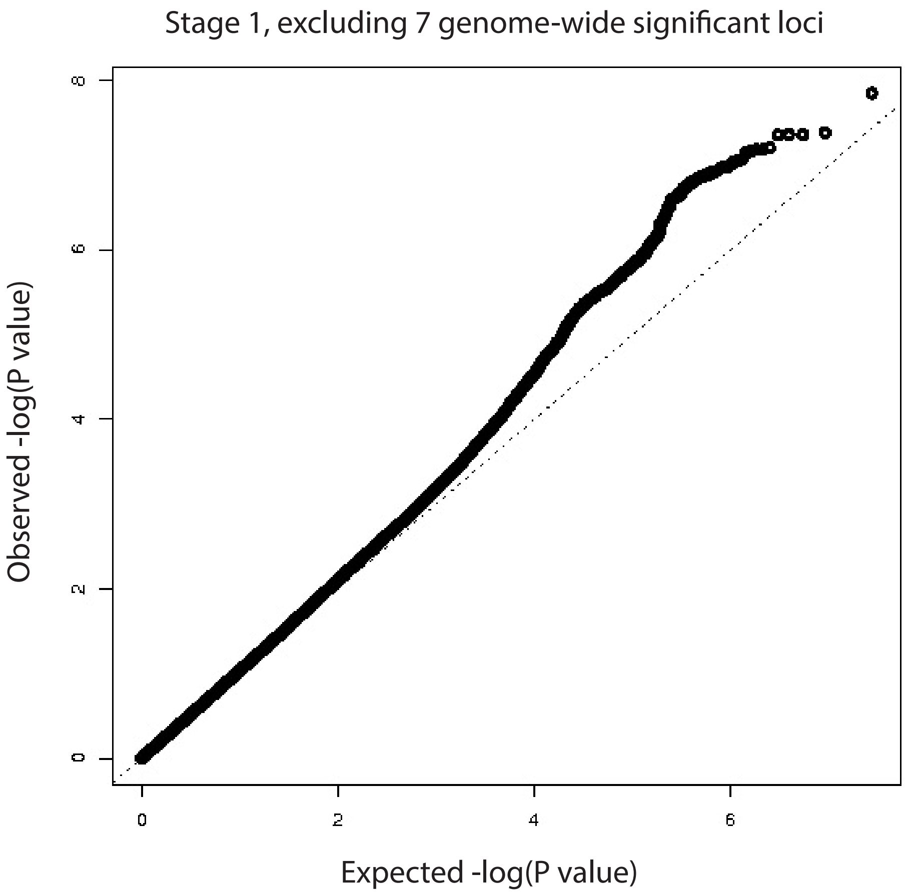

Supplementary Figure 2. QQ plot of observed versus expected quantiles for stage 1 P values, plotted on a log scale, excluding the 7 genome-wide significant loci from stage 1 and their surrounding 1Mb interval. The null hypothesis states that the expected distribution of P values is uniform. The dashed line has a slope 1.

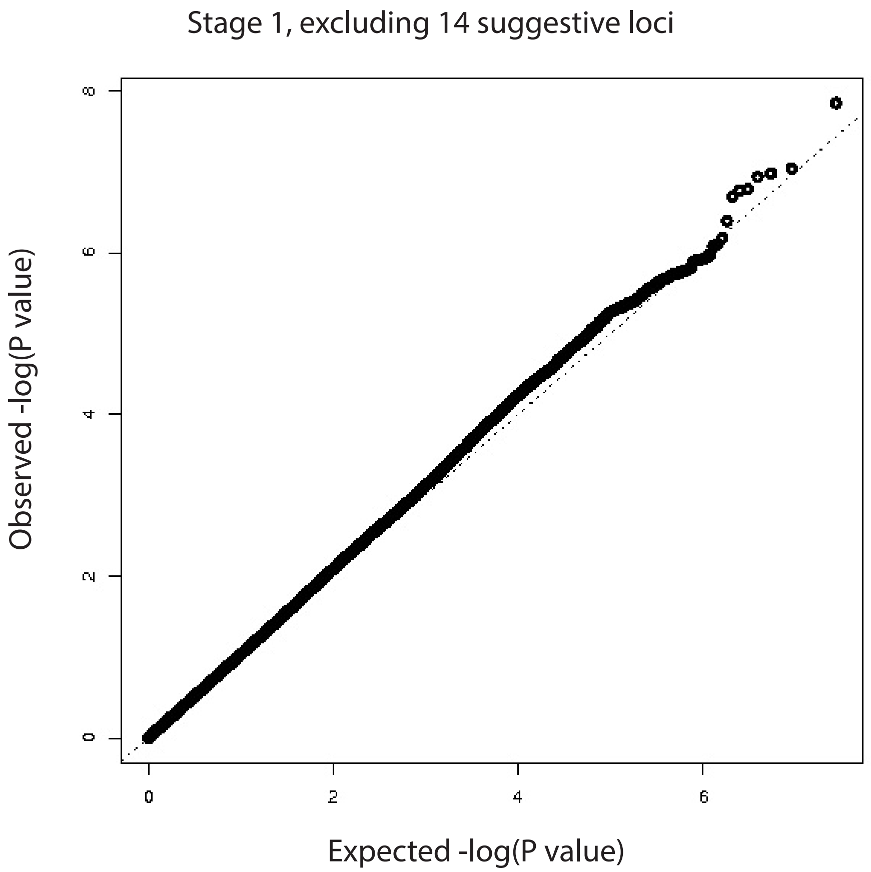

Supplementary Figure 3. QQ plot of observed versus expected quantiles for stage 1 P values, plotted on a log scale, excluding the 14 suggestive loci ($P=1.0x10^{-6}$) and their surrounding 1Mb interval. The null hypothesis states that the expected distribution of P values is uniform. The dashed line has a slope 1.

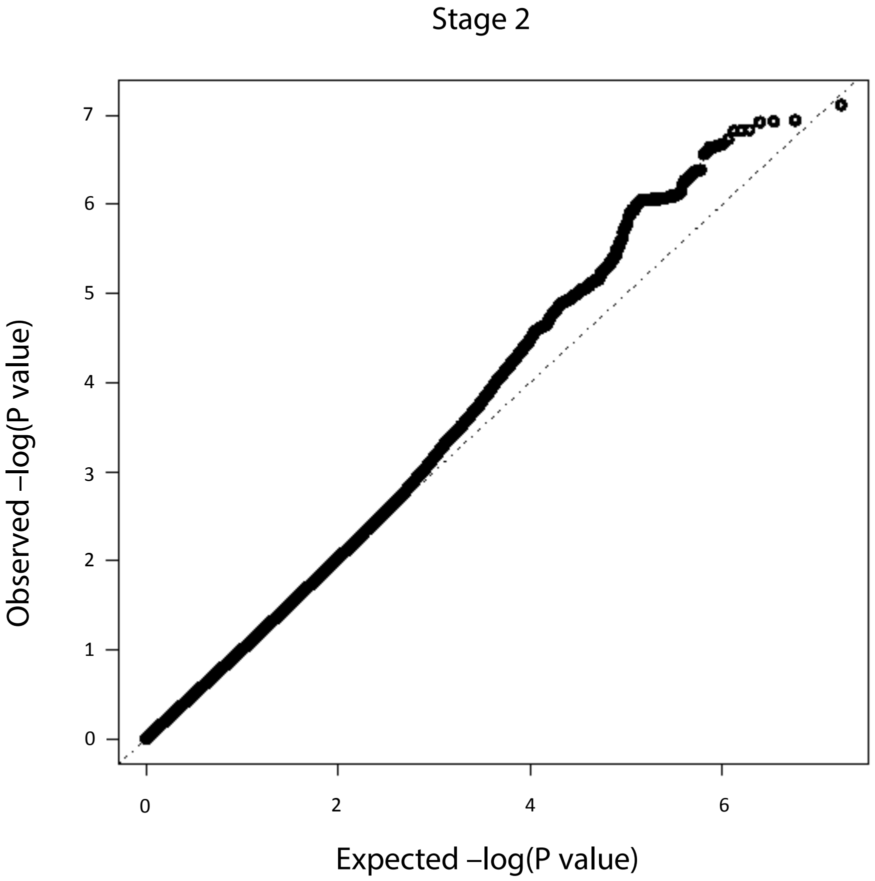

Supplementary Figure 4. QQ plot of observed versus expected quantiles for stage 2 *P* values, plotted on a log scale. The null hypothesis states that the expected distribution of *P* values is uniform. The dashed line has a slope 1.

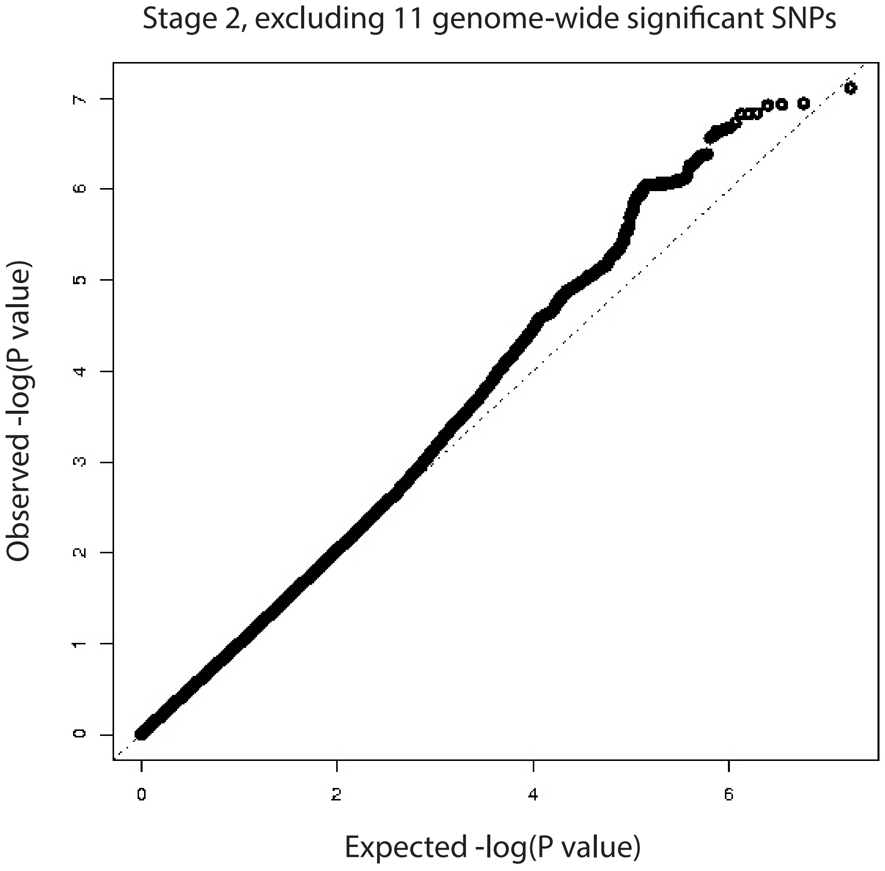

Supplementary Figure 5. QQ plot of observed versus expected quantiles for stage 2 P values, plotted on a log scale, excluding the 11 genome-wide significant SNPs and their surrounding 1Mb intervals. The null hypothesis states that the expected distribution of P values is uniform. The dashed line has a slope 1.

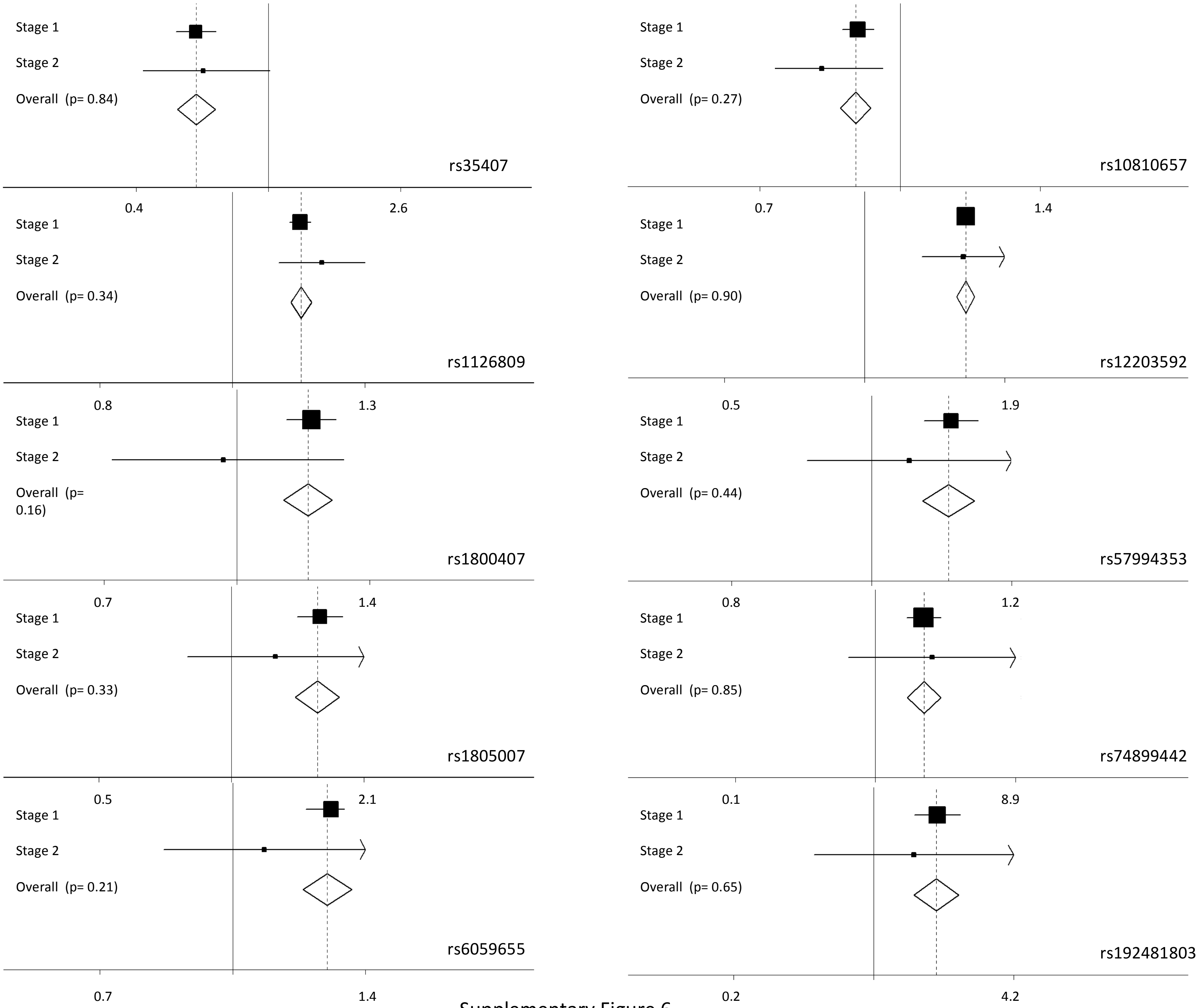

Supplementary Figure 6

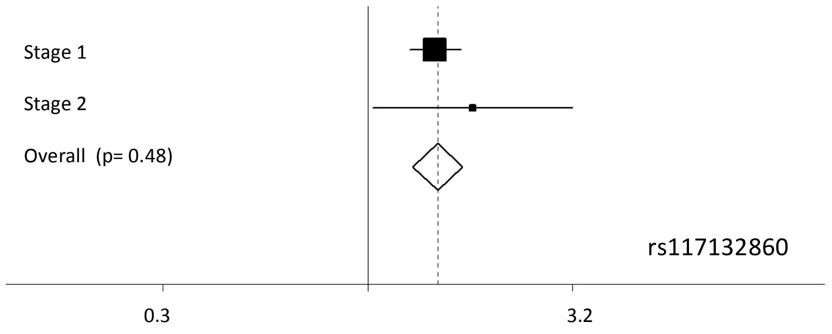

Supplementary Figure 6

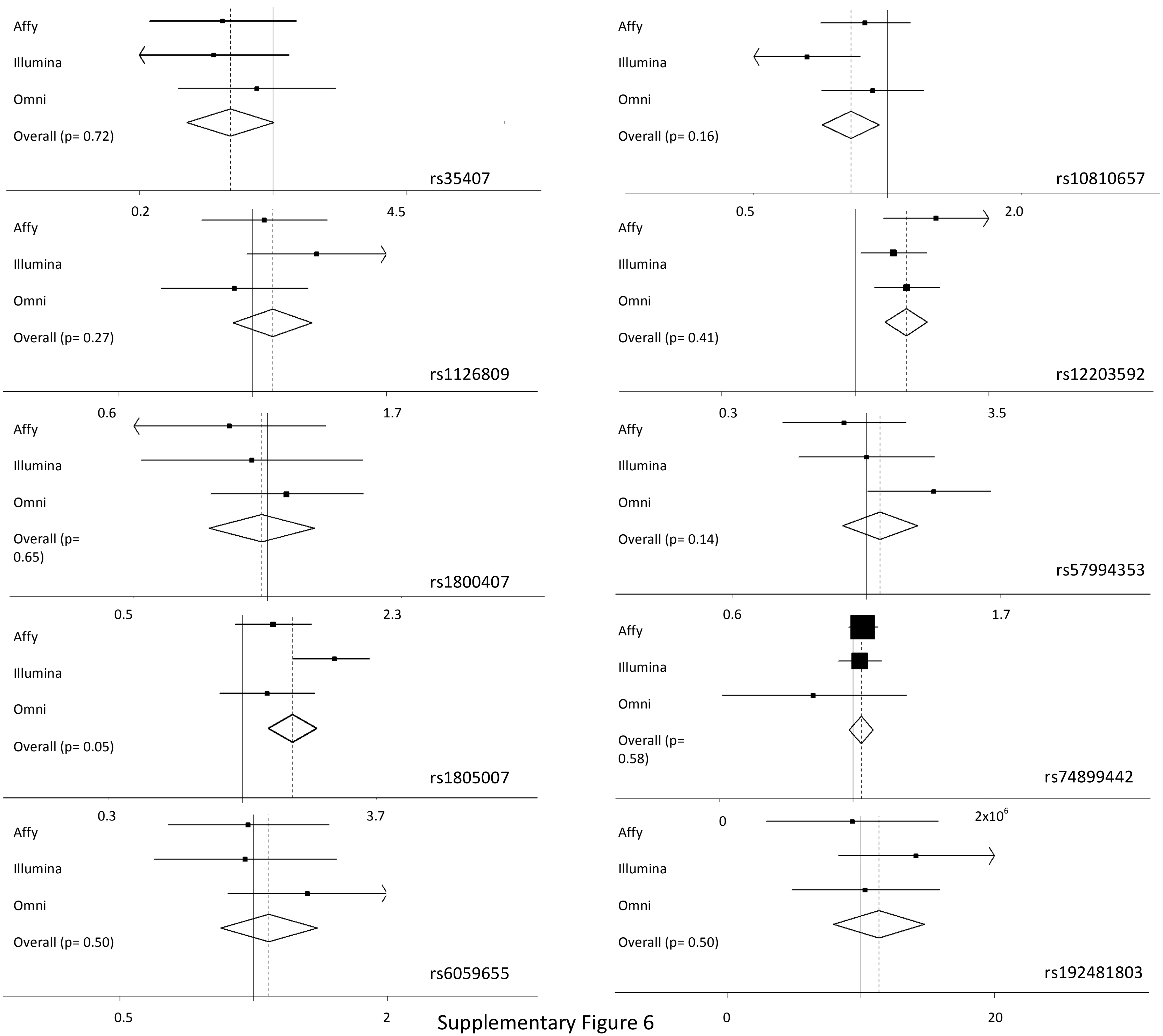

Supplementary Figure 6

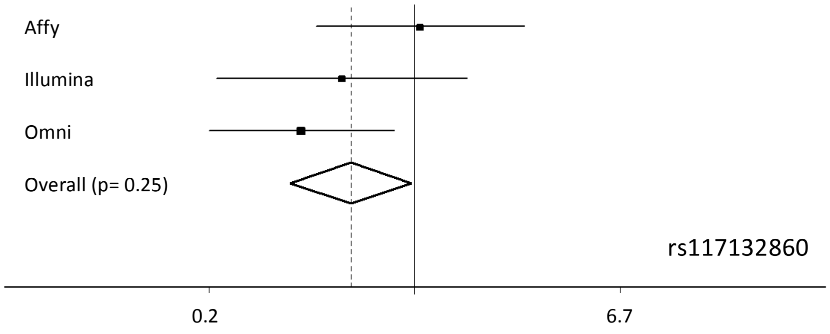

Supplementary Figure 6. Forest plots for each of the 11 SNPs reaching genome-wide significance for association with SCC (via logistic regression). The first set of 11 plots displays odds ratios from stage 1, stage 2, and overall meta-analysis. The second set of 11 plots displays ORs from only stage 2, subdivided by genotyping platform (Affy, Illumina, and Omni) and stage 2 overall. For all plots, x-axis displays odds ratio (OR) values and solid vertical lines represent an odds ratio (OR) of 1. Each dashed vertical line represents the pooled OR estimate from the corresponding meta-analysis, and the diamond represents the 95% CI for this estimate. Black dots indicate OR from each population (see left-most labels) and horizontal black lines represent 95% CI. Black boxes represent precision (only relevant for stage 1 data).

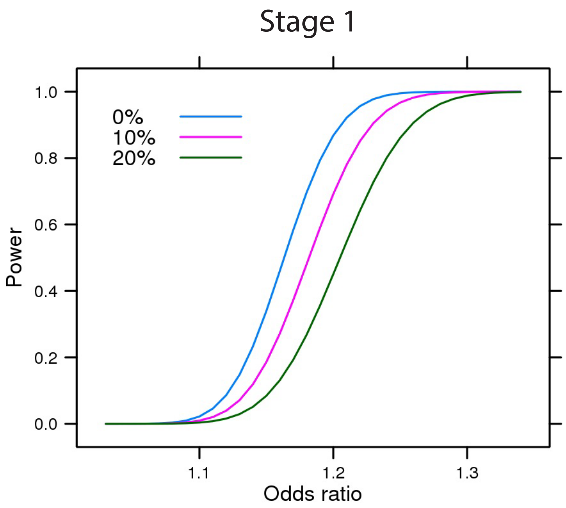

Power	Percent misclassified cases *0%*	*10%*	*20%*
0.2	1.14	1.15	1.17
0.5	1.16	1.18	1.21
0.8	1.19	1.21	1.24

Supplementary Figure 7. Power to detect a variant with misclassifications rates between 0 and 10%. The top graph depicts power as a function of odds ratio for detecting a variant with minor allele frequency 0.1, with misclassification rates of 0%, 10%, and 20% (where the specified fraction of study cases are misclassified controls). The table (bottom) shows odds ratios required to achieve the specified power, for a variant with minor allele frequency 0.1, based on the GWAS sample size, for misclassification rates of 0%, 10%, and 20%. To account for misclassification, expected genotype frequencies in study cases were replaced with a mixture of genotype frequencies in true cases and in true controls.

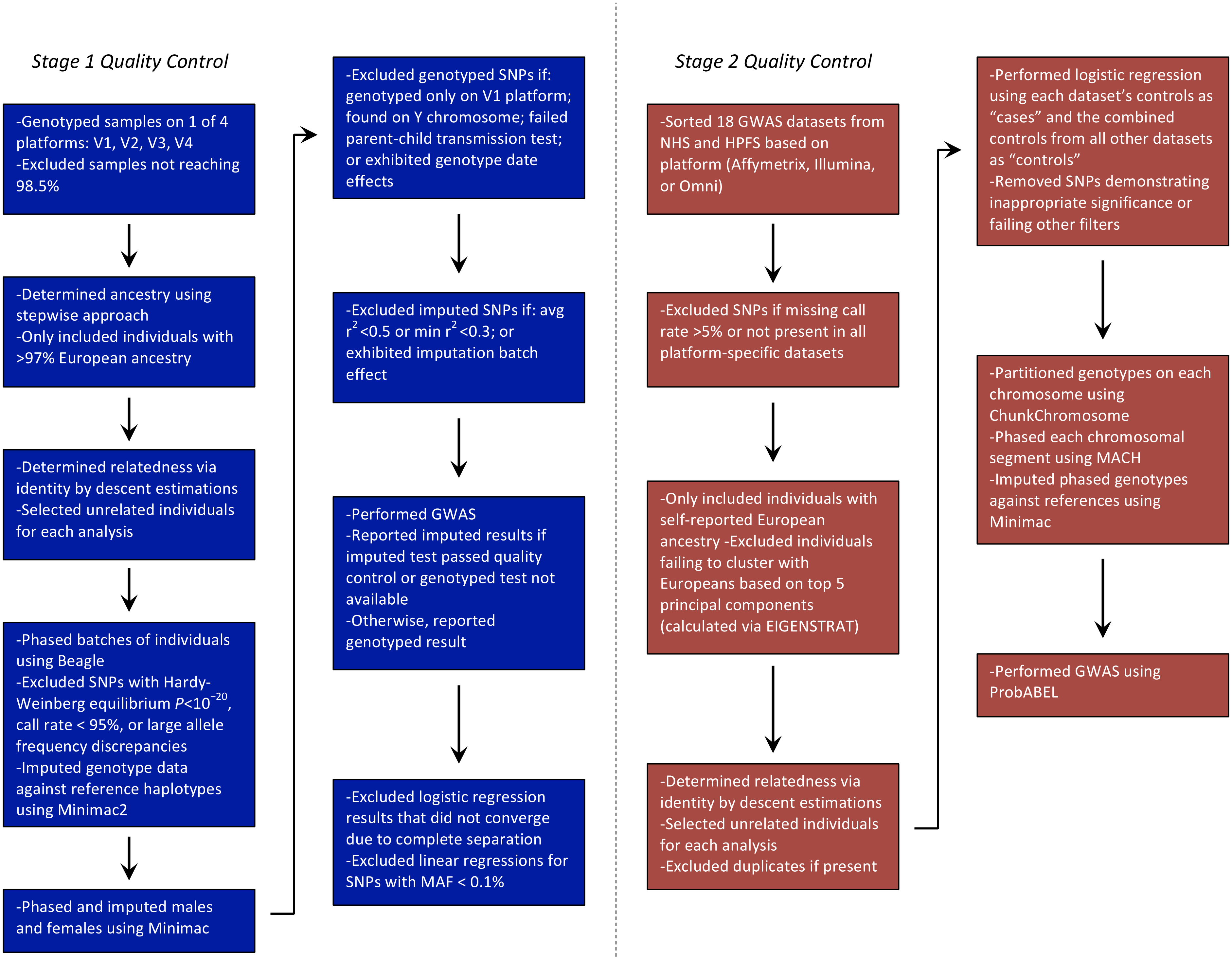

Supplementary Figure 8. Flow chart of quality control for Stage 1 and Stage 2

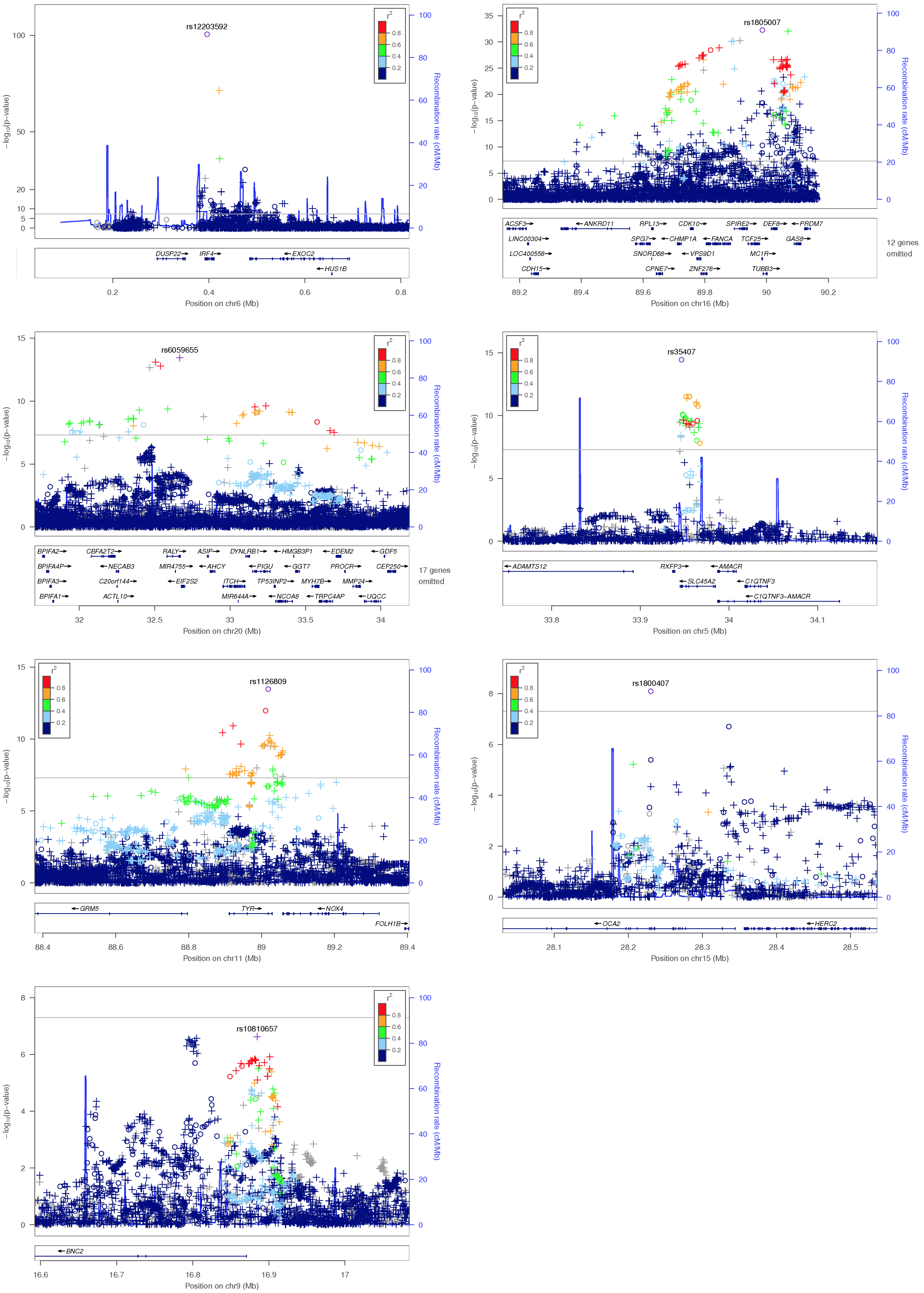

rs12203592
−log10(p−value)
Recombination rate (cM/Mb)
r2
0.8
0.6
0.4
0.2
DUSP22→
IRF4→
←EXOC2
←HUS1B
Position on chr6 (Mb)
rs1805007
ACSF3→
←ANKRD11
RPL13→
CDK10→
SPIRE2→
DEF8→
←PRDM7
LINC00304→
SPG7→
←CHMP1A
←FANCA
TCF25→
GAS8→
LOC400558→
SNORD68→
←VPS9D1
MC1R→
CDH15→
CPNE7→
ZNF276→
TUBB3→
12 genes omitted
Position on chr16 (Mb)
rs6059655
BPIFA2→
CBFA2T2→
RALY→
ASIP→
DYNLRB1→
←HMGB3P1
←EDEM2
←GDF5
BPIFA4P→
←NECAB3
MIR4755→
←AHCY
←PIGU
←GGT7
PROCR→
CEP250→
BPIFA3→
C20orf144→
←EIF2S2
ITCH→
TP53INP2→
MYH7B→
MMP24→
BPIFA1→
ACTL10→
MIR644A→
←NCOA6
←TRPC4AP
←UQCC
17 genes omitted
Position on chr20 (Mb)
rs35407
←ADAMTS12
RXFP3→
←AMACR
←SLC45A2
←C1QTNF3
←C1QTNF3−AMACR
Position on chr5 (Mb)
rs1126809
←GRM5
TYR→
←NOX4
FOLH1B→
Position on chr11 (Mb)
rs1800407
←OCA2
←HERC2
Position on chr15 (Mb)
rs10810657
←BNC2
Position on chr9 (Mb)

Supplementary Figure 9. Regional association plots for 7 of the 11 genome-wide significant SCC susceptibility loci. Each plot is labeled with the rsID for the index SNP corresponding to that locus. These 7 loci are previously confirmed in GWAS. Left to right, beginning from top left: **6p25.3** (rs12203592, in *IRF4*), **16q24.3** (rs1805007, *MC1R* R151C), **20q11.22** (rs6059655, *RALY-ASIP*), **5p13.2** (rs35407, in *SLC45A2*), **11q14.3** (rs1126809, *TYR* R402Q), **15q13.1** (rs1800407, *OCA2* R419Q), **9p22.2** (rs10810657, *BCN2, CNTLN*). Each plot displays -$\log_{10}$(*P* value) versus genomic position based on stage 1 logistic regression association testing. The color scale indicates strength of linkage disequilibrium (r^2) for nearby SNPs, with respect to the index SNP. To preserve detail, results with $P<10^{-100}$ are set to 10^{-100}. The "o" and "+" symbols represent genotyped and imputed SNPs, respectively. Recombination rates, in cM/Mb, are also plotted (navy blue lines). These plots were generated via LocusZoom, using LD data from the March 2012 release of 1000 Genomes data.

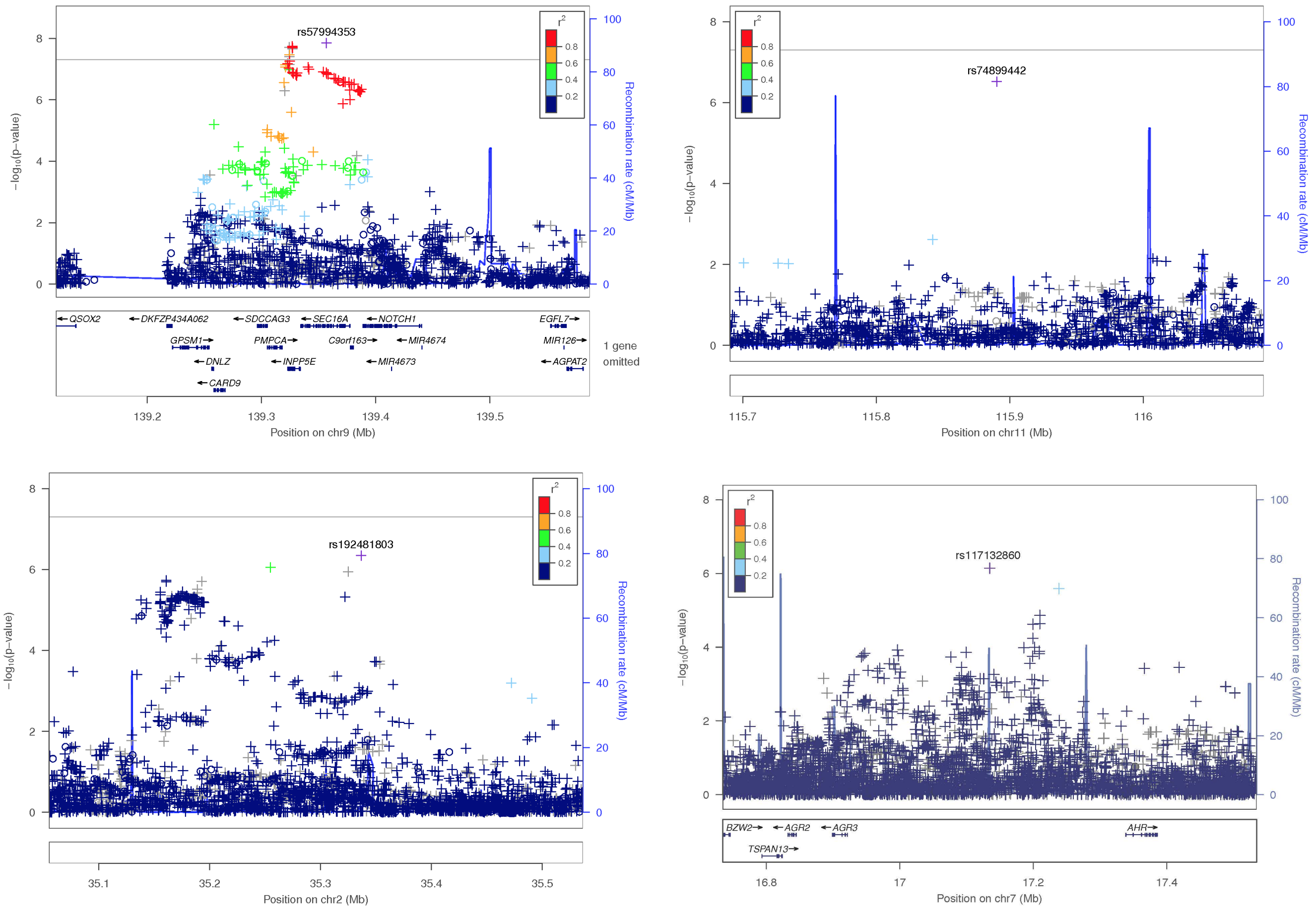

Supplementary Figure 10. Regional association plots for the 4 novel, genome-wide significant SCC susceptibility loci. Each plot is labeled with the rsID for the index SNP corresponding to that locus. These 4 loci are not pigmentation-related. Left to right, beginning from top left: **9q34.3** (rs57994353, *SEC16A*), **11q23.3** (rs74899442, *CADM1, BUD13*), **2p22.3** (rs192481803), **7p21.1** (rs117132860, *AHR*).

Stage 1

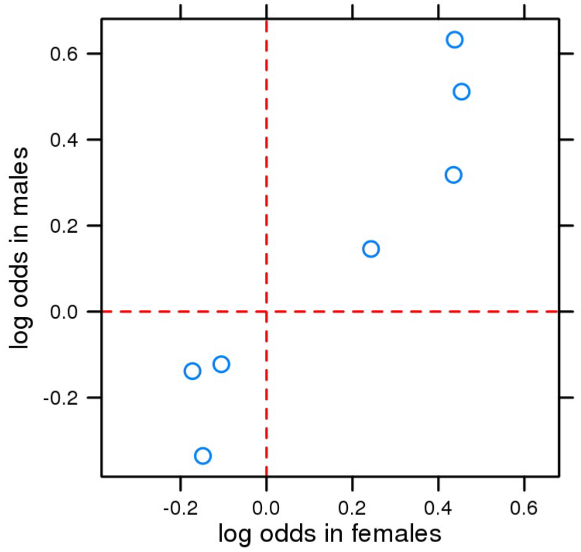

Supplementary Figure 11. Effect sizes estimated in men versus women, for the 7 genome-wide significant loci from stage 1.

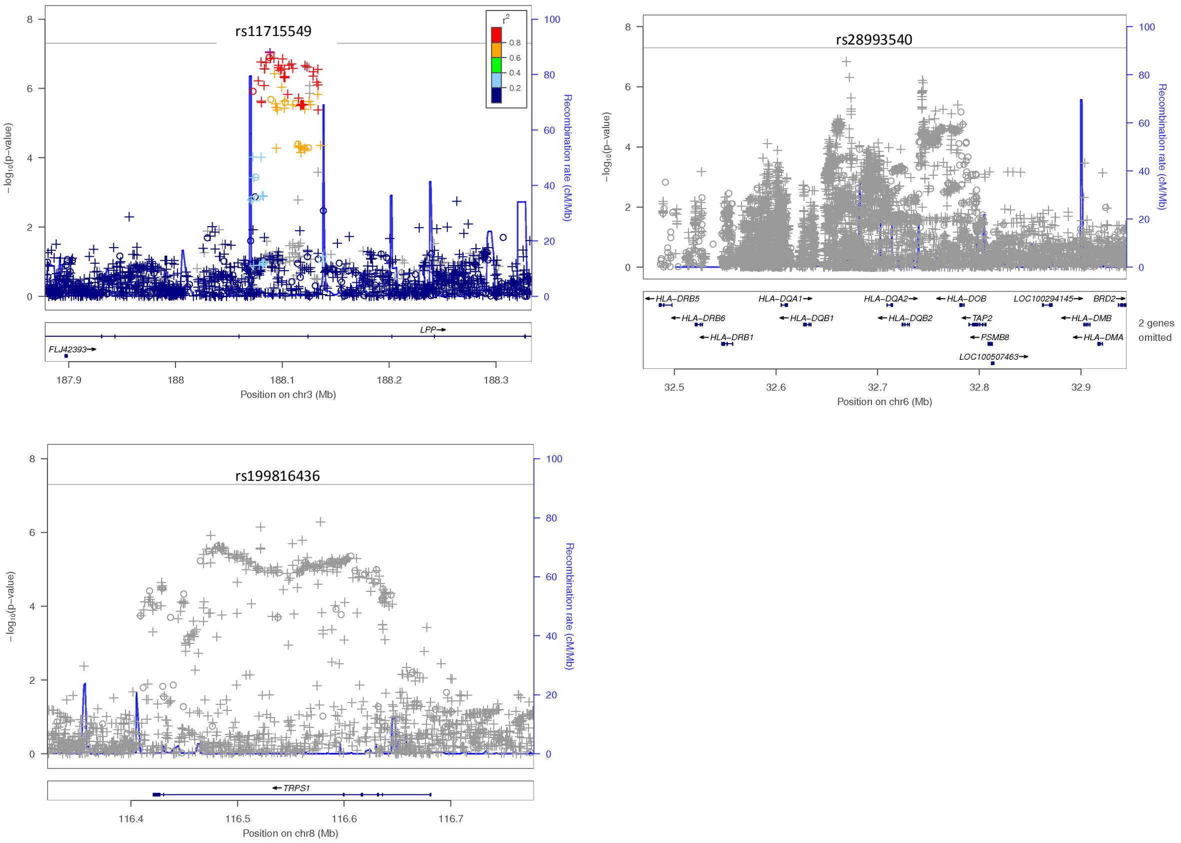

Supplementary Figure 12. Regional association plots for the 3 SCC susceptibility loci that did not reach genome-wide significance. Each plot is labeled with the rsID for the index SNP corresponding to that locus. These 3 loci had $5\times10^{-8} < P < 10^{-6}$ in stage 1 logistic regression analysis. Left to right, beginning from top left: **3q28** (rs11715549, *LPP*), **6p21.32** (rs28993540, *HLA-DQB1*), **8q23.3** (rs199816436, *TRPS1*).

Supplementary Table 1. Sensitivity and specificity of self-report data with respect to SCC diagnosis

n = 188	Disease (+)	Disease (-)
Self-report (+)	24	4
Self-report (-)	2	158
	**Sensitivity = 92%**	
	**Specificity = 98%**	

Within table, from left to right, are counts for true positives, false positives, false negatives, and true negatives. Data from 188 randomly selected patients at Stanford dermatology clinics.

Supplementary Table 2. Imputation and effect heterogeneity statistics for SNPs at 10 previously reported loci

			Stage 1			Stage 2					Meta-analysis	
SNP	Gene	Maj/ min	MAF	avg r^2	min r^2	MAF	Affy r^2	Illum r^2	Omni r^2	Overall avg r^2	P_{het}	I^2 (%)
rs12203592	*IRF4*	C/T	0.17	0.16	0.99	0.16	0.32	1.00	1.00	0.77	0.74	0
rs1805007	*MC1R*	C/T	0.07	0.07	1.00	0.07	1.00	0.82	0.88	0.90	0.36	0
rs35407	*SLC45A2*	G/A	0.04	0.06	0.98	0.06	0.60	0.58	0.33	0.50	0.95	0
rs1126809	*TYR*	G/A	0.28	0.27	0.99	0.27	0.98	0.97	0.96	0.97	0.11	61
rs6059655	*RALY-ASIP*	G/A	0.07	0.07	0.99	0.07	0.98	0.98	0.99	0.98	0.12	59
rs1800407	*OCA2*	C/T	0.07	0.07	1.00	0.07	0.66	0.65	1.00	0.77	0.20	40
rs10810657	*BNC2, CNTLN*	A/T	0.41	0.98	0.97	0.41	0.98	0.97	0.97	0.97	0.19	42
rs62246017	*FOXP1*	G/A	0.33	0.85	0.83	0.32	0.99	0.83	0.82	0.88	0.43	0
rs6791479	*TPRG1/TP63*	A/T	0.43	0.99	0.99	0.43	1.00	0.99	0.99	0.99	0.02	82
rs4455710	*HLA-DQA1*	C/T	-	-	-	-	-	-	-	-	-	-

We report genetic context, major and minor alleles, stage 1 minor allele frequency (MAF), stage 1 average imputation r^2 (avg r^2), stage 1 minimum imputation r^2, stage 2 MAF, stage 2 average imputation r^2 for each genotyping platform (Affy, Illumina, Omni) and overall, and *P* value (P_{het}) and I^2 for effect heterogeneity pertaining to meta-analysis of combined stage 1-stage 2 data.

Supplementary Table 3. Imputation and effect heterogeneity statistics for 4 novel SCC susceptibility loci

			Stage 1			Stage 2					Meta-analysis	
SNP	Gene	Maj/ min	MAF	avg r^2	min r^2	MAF	Affy r^2	Illum r^2	Omni r^2	Overall avg r^2	P_{het}	I^2 (%)
rs57994353	*SEC16A*	T/C	0.301	0.99	0.98	0.299	0.99	0.99	0.99	0.99	0.55	0
rs74899442	*CADM1, BUD13*	T/C	0.004	0.62	0.52	0.005	0.50	0.55	0.56	0.54	0.76	0
rs192481803	*unknown*	C/T	0.007	0.58	0.53	0.007	0.54	0.52	0.57	0.54	0.79	0
rs117132860	*AHR*	G/A	0.023	0.52	0.45	0.02	0.53	0.44	0.51	0.49	0.48	0

We report genetic context, major and minor alleles, stage 1 minor allele frequency (MAF), stage 1 average imputation r^2 (avg r^2), stage 1 minimum imputation r^2, stage 2 MAF, stage 2 average imputation r^2 for each genotyping platform (Affy, Illumina, Omni) and overall, and *P* value (P_{het}) and I^2 for effect heterogeneity pertaining to meta-analysis of combined stage 1-stage 2 data.

Supplementary Table 4. Power to reach *P* <0.05 in stage 2 for all 14 index SNPs

rsSNP	Region	Gene	Power
rs12203592	6p25.3	*IRF4*	0.99
rs1805007	16q24.3	*MC1R*	0.99
rs35407	5p13.2	*SLC45A2*	0.70
rs1126809	11q14.3	*TYR*	0.77
rs6059655	20q11.22	*RALY-ASIP*	0.61
rs1800407	15q13.1	*OCA2*	0.59
rs57994353	9q34.3	*SEC16A*	0.52
rs11715549	3q28	*LPP*	0.50
rs28993540	6p21.32	*HLA-DQB1, HLA-DQA2*	NA
rs10810657	9p22.2	*BNC2, CNTLN*	0.49
rs74899442	11q23.3	*CADM1, BUD13*	0.96
rs192481803	2p22.3	*unknown*	0.88
rs117132860	7p21.1	*AGR3, AHR*	0.64
rs199816436	8q23.3	*TRPS1*	0.48

For each of the index SNPs found in stage 1, we report the estimated power to reach $P < 0.05$ in the stage 2 samples. Four SNPs (6p25.3, 5p13.2, 16q24.3, and 9p22.2) reached $P < 0.05$ in the stage 2 samples.

Supplementary Table 5. Effect size of 7 significant SNPs in stage 1 stratified by age

		Age interval				
**SNP**	**Gene**	(0,60]	(60,67]	(67,73]	(73,73+]	***P-value***
rs12203592	*IRF4*	0.552	0.517	0.449	0.392	0.0087
rs1805007	*MC1R*	0.466	0.433	0.378	0.199	0.0073
rs35407	*SLC45A2*	0.629	0.573	0.414	0.516	0.67
rs1126809	*TYR*	-0.211	-0.113	-0.176	-0.11	0.13
rs6059655	*RALY*	-0.304	-0.216	-0.255	-0.213	0.34
rs1800407	*OCA2*	0.161	0.158	0.211	0.223	0.89
rs57994353	*SEC16A*	-0.078	-0.043	-0.175	-0.157	0.04

The table shows effect sizes (for each SNP reaching genome-wide significance in stage 1) computed in each age interval, as well as the *P* value for the test of genotype interaction with age interval. Stage 1 cohort was divided into four age intervals with similar effective sample sizes based on case and control sample counts. For all these association tests, the same covariates were used as in stage 1: age, sex, and five principal components. Thus, association tests within a specific age interval were still adjusted for age as a continuous covariate. Mean age for the 280,558 controls was 49.2 (SD 16.3); for the 6,579 cases, mean age was 67.1 (SD 11.1).

Supplementary Table 6. Gender-based logistic regression results for significant SNPs in stage 1

SNP	Gene	M_{effect}	M_{SE}	M_P	F_{effect}	F_{SE}	F_P	*P*-value
rs12203592	*IRF4*	0.512	0.030	2.2×10^{-63}	0.454	0.031	1.5×10^{-45}	0.686
rs1805007	*MC1R*	0.318	0.042	1.3×10^{-13}	0.435	0.042	1.7×10^{-23}	0.035
rs35407	*SLC45A2*	0.632	0.103	1.5×10^{-11}	0.438	0.100	2.6×10^{-6}	0.137
rs1126809	*TYR*	-0.138	0.027	4.1×10^{-7}	-0.172	0.029	2.4×10^{-9}	0.256
rs6059655	*RALY*	-0.336	0.042	8.0×10^{-15}	-0.148	0.045	1.3×10^{-3}	0.004
rs1800407	*OCA2*	0.146	0.045	1.6×10^{-3}	0.243	0.045	1.7×10^{-7}	0.080
rs57994353	*SEC16A*	-0.122	0.027	5.4×10^{-6}	-0.105	0.028	2.2×10^{-4}	0.680

The table shows results from logistic regression models (for each SNP reaching genome-wide significance in stage 1) fit separately in male and female subsets of the stage 1 cohort, and a *P*-value from a likelihood ratio test for adding a gender by genotype interaction to the full model. "M" stands for male and "F" for female. The subscripts "SE" and "P" stand for "standard error" and "P-value", respectively. Of the 280,558 controls, 54% were male; of the 6,579 cases, 53% were male.

Supplementary Table 7. Meta-analysis for rs35407 and rs16891982

				Stage 1				Stage 2							Meta-analysis				
SNP	Region	Gene	Maj/ min	*P*	*OR*	95% CI	Avg r^2	*P*	*OR*	95% CI	Affy	Illum	Omni	Avg r^2	*P*	*OR*	95% CI	P_{het}	I^2
rs35407	5p13.2	SLC45A2	G/A	3.6×10^{-15}	0.59	(0.51, 0.68)	0.984	5.5×10^{-2}	0.62	(0.38, 1.01)	0.600	0.578	0.328	0.502	1.3×10^{-13}	0.59	(0.51, 0.68)	0.839	0
rs16891982	5p13.2	SLC45A2	C/G	8.6×10^{-13}	1.65	(1.42, 1.91)	0.995	1.1×10^{-1}	1.42	(0.92, 2.19)	0.561	0.577	0.318	0.485	1.5×10^{-11}	1.62	(1.41, 1.87)	0.524	0

For each SNP, we report rsID, genetic locus, gene, major and minor alleles, *P* value for stage 1 (generated via logistic regression), odds ratio (for stage 1 overall, plus 95% CI), average imputation r^2 (for stage 1), *P* value for stage 2, odds ratio (for stage 2 overall, plus 95% CI), average imputation r^2 (for Affy, Illumina, Omni, stage 2 overall), *P* value for meta-analysis, odds ratio (for meta-analysis overall, plus 95% CI), and P_{het} and I^2 for meta-analysis.

SLC45A2 rs35407 is in modest LD with rs16891982 (Phe374Leu) (r^2=0.33, D'=1). rs16891982 reached genome-wide significance in stage 1 (P = 8.6×10^{-13}, OR = 1.65) and in the overall meta-analysis. We directly genotyped this SNP in a subset of SCC nested case-control study within the NHS[1]. The overall concordance of the SNP rs16891982 between imputed and genotyped data among 300 samples was high (Pearson correlation coefficient *r* of 0.80). We confirmed no material difference in estimates (P = 0.79 for heterogeneity) between imputed data (OR = 2.25) and directly genotyped data (OR = 2.99).

Supplementary Table 8. Meta-analysis for rs12203592 and rs1800407 using subsets with high imputation quality in stage 2

				Stage 2							Meta-analysis				
SNP	Region	Gene	Maj/ min	*P*	*OR*	95% CI	Affy	Illum	Omni	Avg r^2	*P*	*OR*	95% CI	P_{het}	I^2
rs12203592	6p25.3	*IRF4*	C/T	$1.6{\times}10^{-4}$	1.52	(1.22, 1.88)	NA	1.00	1.00	1.00	$4.4{\times}10^{-109}$	1.62	(1.55, 1.69)	0.57	0.0%
rs1800407	15q13.1	*OCA2*	C/T	$6.3{\times}10^{-1}$	1.12	(0.71, 1.76)	NA	NA	1.00	1.00	$1.4{\times}10^{-9}$	1.21	(1.14, 1.29)	0.74	0.0%

For *IRF4* rs12203592, when we limited stage 2 to two datasets with imputation r^2=0.997, the logistic regression results were similar (P = $1.6{\times}10^{-4}$, OR = 1.52) compared to the overall meta-analysis (P = $4.4{\times}10^{-109}$, OR = 1.62). We directly genotyped this SNP in a subset of SCC nested case-control study within the NHS and HPFS (P = $3.2{\times}10^{-8}$, OR = 1.61)[2]. For *OCA2* rs1800407 (Arg419Gln), when we limited our stage 2 to one dataset with imputation r^2=0.996, the results were similar (P = 0.63, OR = 1.12) to the overall meta-analysis (P = $1.4{\times}10^{-9}$, OR = 1.21). We directly genotyped this SNP in a subset of SCC nested case-control study within the NHS (P = 0.07, OR = 1.39)[1].

Supplementary Table 9. Meta-analysis for 4 SNPs within *BNC2,* in linkage disequilibrium with index SNP rs10810657

				Stage 1					Stage 2								Meta-analysis				
SNP	Region	Gene	Maj/ min	MAF	*P*	*OR*	95% CI	Avg r^2	MAF	*P*	*OR*	95% CI	Affy	Illum	Omni	Avg r^2	*P*	*OR*	95% CI	P_{het}	I^2
rs10756819	9p22.2	*BNC2*	A/G	0.34	7.4×10^{-3}	0.95	(0.92, 0.99)	0.999	0.34	6.2×10^{-3}	0.80	(0.68, 0.94)	0.892	0.999	0.999	0.963	1.2×10^{-3}	0.94	(0.91, 0.98)	0.04	76%
rs12350739	9p22.2	*BNC2*	A/G	0.44	4.7×10^{-6}	0.92	(0.89, 0.95)	0.974	0.44	6.9×10^{-3}	0.81	(0.70, 0.95)	0.905	0.966	0.964	0.945	4.6×10^{-7}	0.91	(0.88, 0.95)	0.12	58%
rs62543565	9p22.2	*BNC2*	A/C	0.41	2.0×10^{-6}	0.91	(0.88, 0.95)	0.899	0.40	1.2×10^{-2}	0.82	(0.70, 0.96)	0.893	0.873	0.878	0.882	1.9×10^{-7}	0.91	(0.87, 0.94)	0.19	42%
rs2153271	9p22.2	*BNC2*	C/T	0.42	1.5×10^{-6}	0.91	(0.88, 0.95)	0.999	0.58	1.4×10^{-2}	0.83	(0.72, 0.96)	0.985	0.999	0.999	0.994	5.7×10^{-5}	0.93	(0.90, 0.96)	0.00	92%

For each *BNC2* SNP, we report rsID, genetic locus, gene, major and minor alleles, stage 1 minor allele frequency (MAF), *P* value for stage 1, odds ratio (for stage 1 overall, plus 95% CI), average imputation r^2 (for stage 1), MAF for stage 2, *P* value for stage 2, odds ratio (for stage 2 overall, plus 95% CI), average imputation r^2 (for Affy, Illumina, Omni, stage 2 overall), *P* value for meta-analysis, odds ratio (for meta-analysis overall, plus 95% CI), and P_{het} and I^2 for meta-analysis. *P* values generated via logistic regression.

Supplementary Table 10. Linkage disequilibrium r^2 values between 5 SNPs at 9p22.2 (*BNC2* locus)

	**rs10810657**	rs62543565	rs10756819	rs215327	rs12350739
**rs10810657**	1.00	0.94	0.76	0.97	0.97
rs62543565		1.00	0.67	0.89	0.90
rs10756819			1.00	0.79	0.79
rs215327				1.00	0.97
rs12350739					1.00

Left-most column and top-most row show rsID. Cells contain linkage disequilibrium r^2 values. Bolded SNP is index SNP identified in stage 1.

Supplementary Table 11. Potential SCC susceptibility loci from two-stage GWAS of unrelated Caucasians in the US

					Stage 1		Stage 2		Combined meta-analysis		
SNP	Region	Gene	Major/ minor	MAF (avg imputation r2)	*OR*	*P*	*OR*	*P*	*OR*	*P*	I^2
rs11715549	3q28	*LPP*	C/G	0.46 (1.0)	1.11	$8.7{\times}10^{-8}$	1.03	$6.7{\times}10^{-1}$	1.10	$1.4{\times}10^{-7}$	0%
rs28993540	6p21.32	*HLA-DQB1, HLA-DQA2*	D/I	0.40 (1.0)	1.11	$1.5{\times}10^{-7}$	NA	NA	NA	NA	NA
rs199816436	8q23.3	*TRPS1*	D/I	0.44 (0.90)	1.10	$5.1{\times}10^{-7}$	1.01	$8.6{\times}10^{-1}$	1.10	$1.1{\times}10^{-6}$	18%

SNPs found to be associated with SCC ($P < 10^{-5}$) but not reaching genome-wide significance ($P < 5 \times 10^{-8}$) in stage 1 or overall meta-analysis are listed. Additionally, we report genetic locus, nearest genes, major allele, minor allele, minor allele frequency (MAF) in stage 1, average imputation r^2 (a measure of imputation quality) for stage 1, and odds ratio (OR) with *P* value for each stage, calculated with respect to the minor allele. I^2 statistic for effect heterogeneity is also included. *P* values generated via logistic regression.

Supplementary references

1. Nan H, Kraft P, Hunter DJ, Han J. Genetic variants in pigmentation genes, pigmentary phenotypes, and risk of skin cancer in Caucasians. *Int J Cancer* **125**, 909-917 (2009).

2. Han J*, et al.* A germline variant in the interferon regulatory factor 4 gene as a novel skin cancer risk locus. *Cancer Res* **71**, 1533-1539 (2011).
